# Incidence of Cancer Among Adults With Thrombocytosis in Ontario, Canada

**DOI:** 10.1001/jamanetworkopen.2021.20633

**Published:** 2021-08-12

**Authors:** Vasily Giannakeas, Steven A. Narod

**Affiliations:** 1 Women’s College Research Institute, Women’s College Hospital, Toronto, Ontario, Canada; 2 Dalla Lana School of Public Health, University of Toronto, Toronto, Ontario, Canada; 3 ICES, Toronto, Ontario, Canada; 4 Institute of Medical Science, University of Toronto, Toronto, Ontario, Canada

## Abstract

**Importance:**

Individuals with newly diagnosed cancer often have a high platelet count (thrombocytosis). Whether thrombocytosis is associated with the presence of an undiagnosed cancer remains unknown.

**Objective:**

To assess whether a new diagnosis of thrombocytosis is associated with a subsequent risk of cancer among adults.

**Design, Setting, and Participants:**

This population-based retrospective cohort study was conducted using linked laboratory data from Ontario, Canada, from January 1, 2007, to December 31, 2017, with follow-up until December 31, 2018. The study cohort included adults aged 40 to 75 years on the date of a routine complete blood count (CBC) test (index test) who had a normal platelet count in the 2 previous years and no history of cancer. Data analysis was performed in December 2020.

**Exposures:**

Exposed individuals were those with a platelet count greater than 450 × 10^9^/L. Matched unexposed control individuals had a platelet count within the reference range (150 × 10^9^/L to 450 × 10^9^/L) reported within 30 days of the exposure.

**Main Outcomes and Measures:**

Incident cancers within 5 years after diagnosis of thrombocytosis. Absolute and relative risks for cancer associated with thrombocytosis were estimated for all cancers and for cancers at specific sites.

**Results:**

Of the 3 386 716 Ontario residents with a recorded routine CBC test result, 53 339 (1.6%) had thrombocytosis and a prior normal platelet count. Among individuals with thrombocytosis, the median age was 59.7 years (interquartile range, 50.2-67.4 years) and 37 349 (70.0%) were women. Among the 51 624 individuals with thrombocytosis included in the matched analysis, 2844 (5.5%) had received a diagnosis of a solid cancer in the 2-year follow-up period and 3869 (7.5%) had received a diagnosis within 5 years. The relative risk (RR) for developing any solid cancer within 2 years was 2.67 (95% CI, 2.56-2.79). Associations were found between thrombocytosis and cancers of the ovary (RR, 7.11; 95% CI, 5.59-9.03), stomach (RR, 5.53; 95% CI, 4.12-7.41), colon (RR, 5.41; 95% CI, 4.80-6.10), lung (RR, 4.41; 95% CI, 4.02-4.85), kidney (RR, 3.64; 95% CI, 2.94-4.51), and esophagus (RR, 3.64; 95% CI, 2.46-5.40).

**Conclusions and Relevance:**

In this cohort study, an increased platelet count was associated with an increased risk of cancer for at least 2 years. The results suggest that individuals with unexplained thrombocytosis should be offered screening for several cancers.

## Key Points

**Question:**

Is a high platelet count associated with an increased risk of cancer among adults?

**Findings:**

In this Canadian population-based cohort study of 53 339 adults aged 40 to 75 years who had received a diagnosis of thrombocytosis based on routine complete blood count test findings and had a normal platelet count in the prior 2 years, thrombocytosis was associated with an increased risk of cancer.

**Meaning:**

The findings suggest that adults with newly identified thrombocytosis may be at increased risk of several types of cancer and should be screened appropriately.

## Teaser

This cohort study examines the association between a new diagnosis of thrombocytosis and the subsequent incidence of cancer among adults aged 40 to 75 years in Ontario, Canada.

## Introduction

The discovery that spontaneous thrombosis was often followed by a diagnosis of cancer was made in 1865 by Armand Trousseau.^1^ The onset of venous thromboembolism has been seen with several types of cancer.^2,3,4^ Patients with newly diagnosed cancer often have concomitant thrombocytosis, which is typically defined as a platelet count greater than 450 × 10^9^/L (to convert to ×10^3^/μL, divide by 1.0).^5^

Platelets are thought to have a role in carcinogenesis and cancer progression, including angiogenesis and metastases.^6,7,8^ It has been shown that some ovarian cancers produce interleukin 6, which in turn upregulates platelet formation in the liver through local production of thrombopoietin.^9^

An increased platelet count has been shown to be associated with the risk of future cancers.^10,11,12^ Whether tumors induce increased platelet formation or whether high platelet counts accelerate the growth of an existing cancer is unclear. Furthermore, it is not known whether the association between a high platelet count and cancer is transient or a prolonged phenomenon. The range of cancers associated with an increased platelet count remains to be determined.

Thrombocytosis may be attributable to blood loss or inflammation, but many cases of thrombocytosis are found at the time of a routine blood test and are unexplained.^13^ We observed a large cohort of adult residents of Ontario, Canada, who had 1 or more routine blood tests with a normal platelet count. Individuals who subsequently developed thrombocytosis in the accrual period were matched to control individuals with platelet counts in the reference range. We observed them for 5 years thereafter to investigate whether thrombocytosis was associated with risk of cancer overall and by tumor site.

## Methods

### Study Design, Population, and Data

We performed a retrospective cohort study of the association between a high platelet count (thrombocytosis) and the incidence of cancer. We extracted information on all routine complete blood count (CBC) tests conducted in Ontario between January 1, 2007, and December 31, 2017, that were available in the provincial laboratory data set. We used the provincial cancer registry to capture all reported cancers diagnosed up to December 31, 2018. Data analysis was performed in December 2020. Data were made available to us through ICES, a nonprofit organization that maintains a centralized repository of health care and demographic data that can be used by scientists for research purposes. ICES is a prescribed entity under §45 of Ontario’s Personal Health Information Protection Act, which allows for research conduct without review by a research ethics board. This study followed the Strengthening the Reporting of Observational Studies in Epidemiology (STROBE) reporting guideline.

Ontario has a universal health insurance program that is publicly funded for all 14.5 million residents. Recently, the Ontario Laboratory Information System database, which contains laboratory testing data obtained since January 2007, was linked with ICES. Included in this data set are 85 868 893 CBC measurements, which represent 11 190 990 Ontario residents. The Ontario Cancer Registry has records of all incident cancer diagnoses in Ontario between January 1964 and December 2018.

Additional data sources of information included physician billing data, emergency department visits, acute care hospitalizations, and patient rosters of primary care practitioners. These data sets were linked using unique encoded identifiers at ICES. Details of all data sets used are available in eTable 1 in the Supplement.

### Construction of the Cohort

Using laboratory data, we identified all Ontario residents aged 40 to 75 years who had 1 or more routine CBC tests performed between January 1, 2007, and December 31, 2017. A CBC test was considered routine if it was ordered by a medical doctor in a community practice setting with a standard priority status (ie, it was not expedited). Individuals were added to the study cohort based on the date of their first eligible CBC test. We excluded CBC tests from individuals who had a history of any cancer recorded in the provincial cancer registry. To ensure that included individuals had a history of platelet levels in the reference range, we also excluded CBC tests from individuals who had no recent history of a routine CBC test (9 to 24 months before the index CBC test) and from those who had any CBC test result recorded in the prior 2 years with a platelet value outside the reference range (eFigure 1 in the Supplement).

### Exposure Definition

The exposed group included patients followed up from their date of cohort entry to a first record indicating thrombocytosis (>450 × 10^9^ platelets/L) from a routine CBC test ordered by a physician in a community setting. The index date for patients in the exposed group was the date of the first CBC test indicating thrombocytosis.

**Figure 1.**
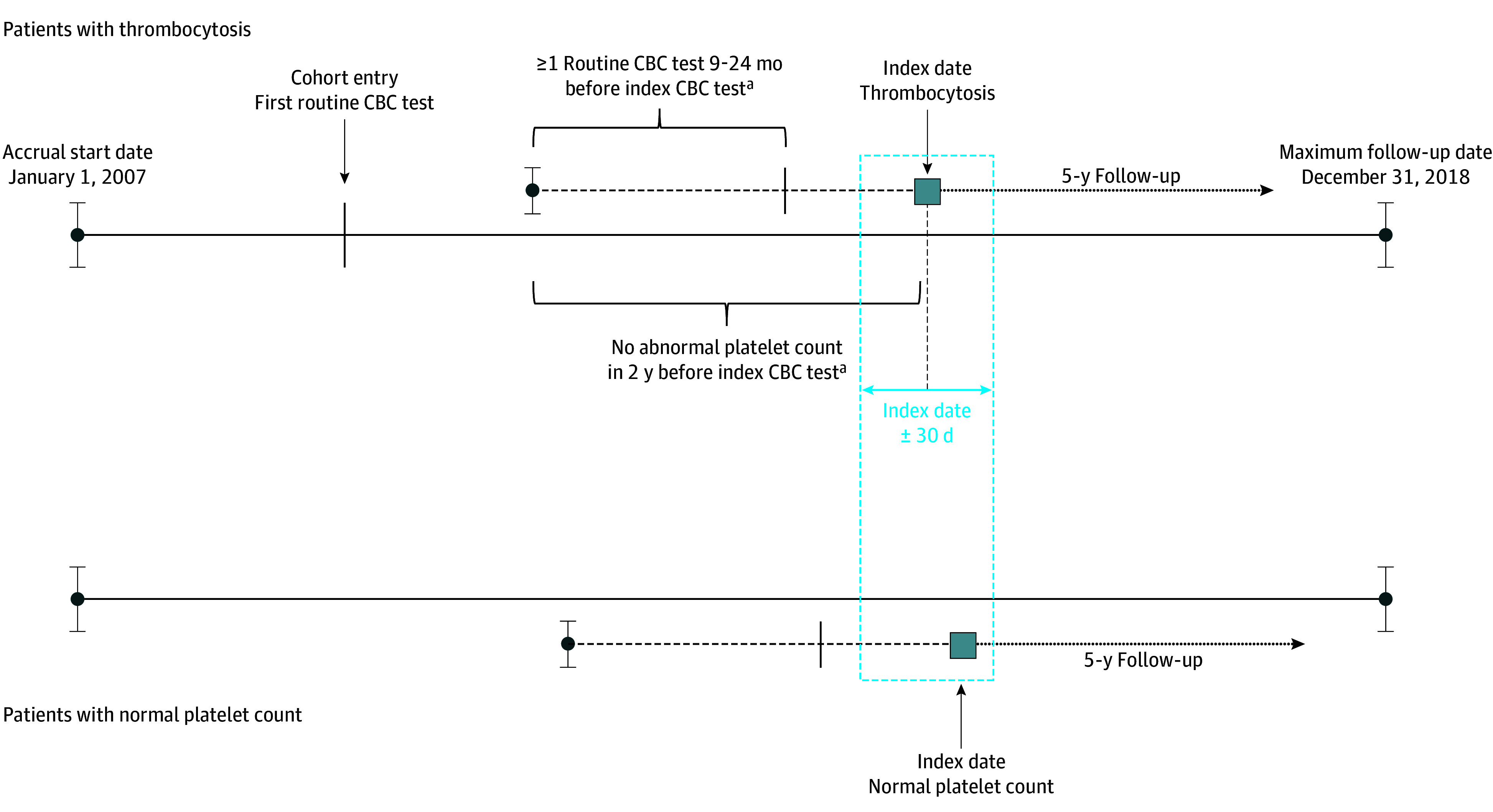
Study Design Criteria Among Matched Individuals A normal platelet count was defined as between 150 × 10^9^/L and 450 × 10^9^/L (to convert to ×10^3^/μL, divide by 1.0). ^a^Condition applied to both patients with thrombocytosis and those with a normal platelet count.

Each exposed individual was matched to 5 unexposed control individuals who had a routine CBC test with a platelet count in the reference range (defined as between 150 × 10^9^ and 450 × 10^9^ platelets/L) within 30 days of the exposure (Figure 1). The index date for the unexposed controls was the date of their routine CBC test. Exposed individuals were matched 1:5 to unexposed controls on the basis of age (rounded to the nearest integer) and sex and were caliper matched based on propensity score, which incorporated demographic variables and health conditions (details are provided in eTable 5 in the Supplement). Patients who developed thrombocytosis were eligible to serve as unexposed controls if they had prior CBC records with platelet counts in the reference range.

### Baseline Variables

Baseline variables at the time of collection of blood samples for the CBC test were measured for descriptive purposes and to ensure balance after matching. We included general demographic information (sex, age, income, and residence), use of primary care services, and comorbidities and chronic conditions. Detailed definitions of variables and the corresponding validation studies are provided in eTable 2 in the Supplement.

### Outcome Definition

Incident cancers were classified into solid and hematologic subtypes based on the site-specific and morphologic values (eTable 3 in the Supplement). Hematologic subtypes included leukemias, lymphomas, multiple myeloma, and other hematologic tumors. Matched members of the cohort were observed for the earliest incident cancer diagnosis from the index date until loss of eligibility of provincial health insurance coverage, death, 5 years of follow-up, or the end of the follow-up period (December 31, 2018).

### Statistical Analysis

We estimated the absolute and relative risks of cancer during the 5-year period after the index date. The absolute risk referred to the total number of cancers observed in members of the cohort during the 5-year period after the index date per 1000 individuals at risk at the beginning of the observation period. The risk was calculated for the entire 5-year follow-up and for individual periods during follow-up (0 to 6 months, >6 to 12 months, >1 to 2 years, >2 to 3 years, >3 to 4 years, and >4 to 5 years). The risks were calculated for exposed (thrombocytosis) and unexposed (normal platelet count) individuals by age group (40-49, 50-59, or 60-75 years) and by sex. Risks were calculated for all cancers, solid tumors, and hematologic tumors and for 18 different types of solid tumors including colon, lung, breast (females only), ovary, cervix, endometrium, prostate, thyroid, pancreas, stomach, kidney, bladder, esophagus, other gastrointestinal, brain, melanoma, head and neck, and other solid tumor. Relative risks were estimated as the ratio of the risk in the exposed group (thrombocytosis) to the risk in the unexposed control individuals for the period being considered. Wald confidence limits were estimated for risk proportions, relative risks, and risk differences, and *z* scores were calculated and applied to 2-sided equality tests for relative risks and risk differences. Significance was set at *P* < .05. Data were analyzed using SAS, version 9.4 (SAS Institute Inc).

We performed 2 sensitivity analyses to explore associations under different design conditions. First, we measured the association of a high platelet count with cancer using an alternate cutoff of 400 × 10^9^/L for thrombocytosis (and between 150 × 10^9^/L and 400 × 10^9^/L for a reference platelet count). In the second sensitivity analysis, we restricted the assessment of the association between a high platelet count and cancer to the results of routine blood tests done in a family practice setting. For this analysis, we restricted the data to CBC tests ordered by a patient’s family doctor. Both analyses required rematching of exposed to unexposed patients using the same procedure as in the initial match.

## Results

Of the 3 386 716 Ontario residents with a recorded routine CBC test result, 53 339 (1.6%) had thrombocytosis (37 349 [70.0%] women and 15 990 [30.0%] men) and were eligible for the cohort study (ie, all had a recent platelet count within the reference range). The median age at the time of the index CBC test indicating thrombocytosis was 59.7 years (interquartile range [IQR], 50.2-67.4 years). Descriptive statistics of all eligible individuals are provided in eTable 4 in the Supplement.

Among individuals with thrombocytosis, 51 624 (96.8%) were successfully matched to 5 controls with normal platelet counts. A balance of demographic data, primary care services, and comorbidities was retained across matched individuals (eTable 5 in the Supplement). The mean platelet count among matched individuals with thrombocytosis was 510 × 10^9^/L (range, 451-2010 × 10^9^/L). The mean age at the index date was 58.8 years (range, 40-75 years). The median number of routine CBC tests in the 2 years before the index date was 2 (IQR, 1-3). A total of 86.8% of individuals in the matched cohort were on the patient roster of a family doctor. Among these individuals, 76.4% of the index CBC tests were ordered by the patient’s family doctor.

During the 5-year period after the index date, 5008 incident cancers were reported among the patients with thrombocytosis (97 cases per 1000 individuals), including 3869 solid tumors (74.9 per 1000 individuals) and 1139 hematologic cancers (22.1 per 1000 individuals). During the 5-year period after the index date, 11 679 incident cancers were reported among the controls (45.2 cases per 1000 individuals), including 10 265 solid tumors (39.8 per 1000 individuals) and 1414 hematologic cancers (5.5 per 1000 individuals).

Most solid cancers were diagnosed within the first 2 years after the index date. Among the 51 624 individuals with thrombocytosis, 2844 (5.5%) had received a diagnosis of a solid cancer in the 2-year follow-up period and 3869 (7.5%) had received a diagnosis within 5 years. Among individuals with thrombocytosis, 5.6% of men and 3.9% of women were diagnosed with an incident cancer within 1 year of the index date. Overall, 7.1% (95% CI, 6.6%-7.5%) of the men with thrombocytosis and 4.9% (95% CI, 4.6%-5.1%) of the women with thrombocytosis received a diagnosis of a solid cancer within 2 years of the index date. The risk was 1.9% (95% CI, 1.7%-2.2%) among those aged 40 to 49 years, 4.5% (95% CI, 4.1%-4.8%) among those aged 50 to 59 years, and 7.8% (95% CI, 7.5%-8.2%) among those aged 60 to 75 years. A total of 2.1% (95% CI, 1.9%-2.3%) of the men with thrombocytosis and 1.2% (95% CI, 1.1%-1.3%) of the women with thrombocytosis received a diagnosis of a hematologic cancer within 2 years of the index date.

The relative risk (RR) for developing a hematologic cancer during the 5-year follow-up period among those with thrombocytosis vs controls was 4.03 (95% CI, 3.73-4.35). The RR for developing any solid tumor during the entire 5-year follow-up period was 1.88 (95% CI, 1.82-1.95); during the first 2 years, 2.67 (95% CI, 2.56-2.79); and during years 3 to 5, 1.10 (95% CI, 1.03-1.18). The RR for all solid tumors in the interval from 3 months to 1 year after the index date was 1.99 (95% CI, 1.83-2.17). The RR for all solid tumors occurring within 2 years of the index date was 2.66 (95% CI, 2.48-2.86) among men and 2.68 (95% CI, 2.53-2.83) among women. The RR was 2.42 (95% CI, 2.08-2.82) among those aged 40 to 49 years, 3.21 (95% CI, 2.90-3.54) among those aged 50 to 59 years, and 2.57 (95% CI, 2.44-2.71) among those aged 60 to 75 years.

The 2-year RRs were most pronounced for ovarian cancer (RR, 7.11; 95% CI, 5.59-9.03), for which 0.4% of female patients with thrombocytosis received a diagnosis within 2 years; the RR for developing ovarian cancer in the first 6 months was very high (23.33; 95% CI, 15.73-34.61). The 2-year RRs were also pronounced for stomach cancer (RR, 5.53; 95% CI, 4.12-7.41), colon cancer (RR, 5.41; 95% CI, 4.80-6.10), lung cancer (RR, 4.41; 95% CI, 4.02-4.85), kidney cancer (RR, 3.64; 95% CI, 2.94-4.51), and esophageal cancer (RR, 3.64; 95% CI, 2.46-5.40). No increased risk was observed for breast cancer (RR, 0.96; 95% CI, 0.82-1.12), prostate cancer (RR, 0.94; 95% CI, 0.78-1.14), or thyroid cancer (RR, 1.05; 95% CI, 0.80-1.37) (eTable 6 in the Supplement). The absolute risk of ovarian cancer in the first 2 years after a diagnosis of thrombocytosis was 4.5 per 1000 individuals, and the absolute risk of breast cancer was 5.2 per 1000 individuals (Table).

**Table.** Probability of Having an Incident Cancer Within 2 Years of the Index Complete Blood Count Test by the Most Common Sites of Solid Cancer^a^

**Cancer site**	**Risk per 1000 individuals **	**95% CI**
**Men**		
With thrombocytosis		
Lung	20.4	18.2-22.7
Colon	14.4	12.5-16.3
Prostate	7.9	6.5-9.4
Kidney	4.7	3.6-5.7
Stomach	3.4	2.5-4.3
Bladder	2.8	2.0-3.7
All solid tumors	70.5	66.5-74.6
With normal platelet count		
Prostate	8.4	7.8-9.1
Lung	4.4	3.9-4.8
Colon	2.7	2.3-3.1
Bladder	2.0	1.7-2.3
Head and neck	1.4	1.2-1.7
Melanoma	1.1	0.9-1.3
All solid tumors	26.5	25.4-27.6
**Women**		
With thrombocytosis		
Lung	13.6	12.4-14.8
Colon	9.2	8.3-10.2
Breast	5.2	4.5-5.9
Ovary	4.5	3.8-5.1
Endometrium	2.3	1.8-2.8
Kidney	2.0	1.6-2.5
All solid tumors	48.6	46.4-50.8
With normal platelet count		
Breast	5.4	5.1-5.8
Lung	3.2	2.9-3.4
Colon	1.7	1.5-1.9
Endometrium	1.5	1.3-1.6
Thyroid	1.4	1.3-1.6
Melanoma	0.7	0.6-0.8
All solid tumors	18.2	17.6-18.8

^a^ The index test for patients in the exposed group (with thrombocytosis) was the date of the first complete blood cell test indicating thrombocytosis. For the unexposed control group (with normal platelet count), the index test could be any CBC in the observation period that met the criteria of an unexposed individual.

The relative risk of pancreatic cancer was 3.75 (95% CI 2.58-5.45) during the first 6 months after a diagnosis of thrombocytosis but was 1.34 (95% CI, 1.01-1.78) from 6 months to 5 years after the diagnosis. The relative risk of esophageal cancer was 9.12 (95% CI, 5.05-16.47) during the first 6 months after a diagnosis of thrombocytosis but was 1.49 (95% CI, 0.95-2.35) from 6 months to 5 years after the diagnosis. A high platelet count was associated with greater RRs for kidney and cervical cancer during the first 6 months after diagnosis of thrombocytosis (kidney: RR, 8.31 [95% CI, 6.06-11.38]; cervix: RR, 3.53 [95% CI, 1.69-7.39]); however, the absolute risk of these cancers was small (kidney, 2.81 per 1000 individuals; cervix, 0.47 per 1000).

**Figure 2.**
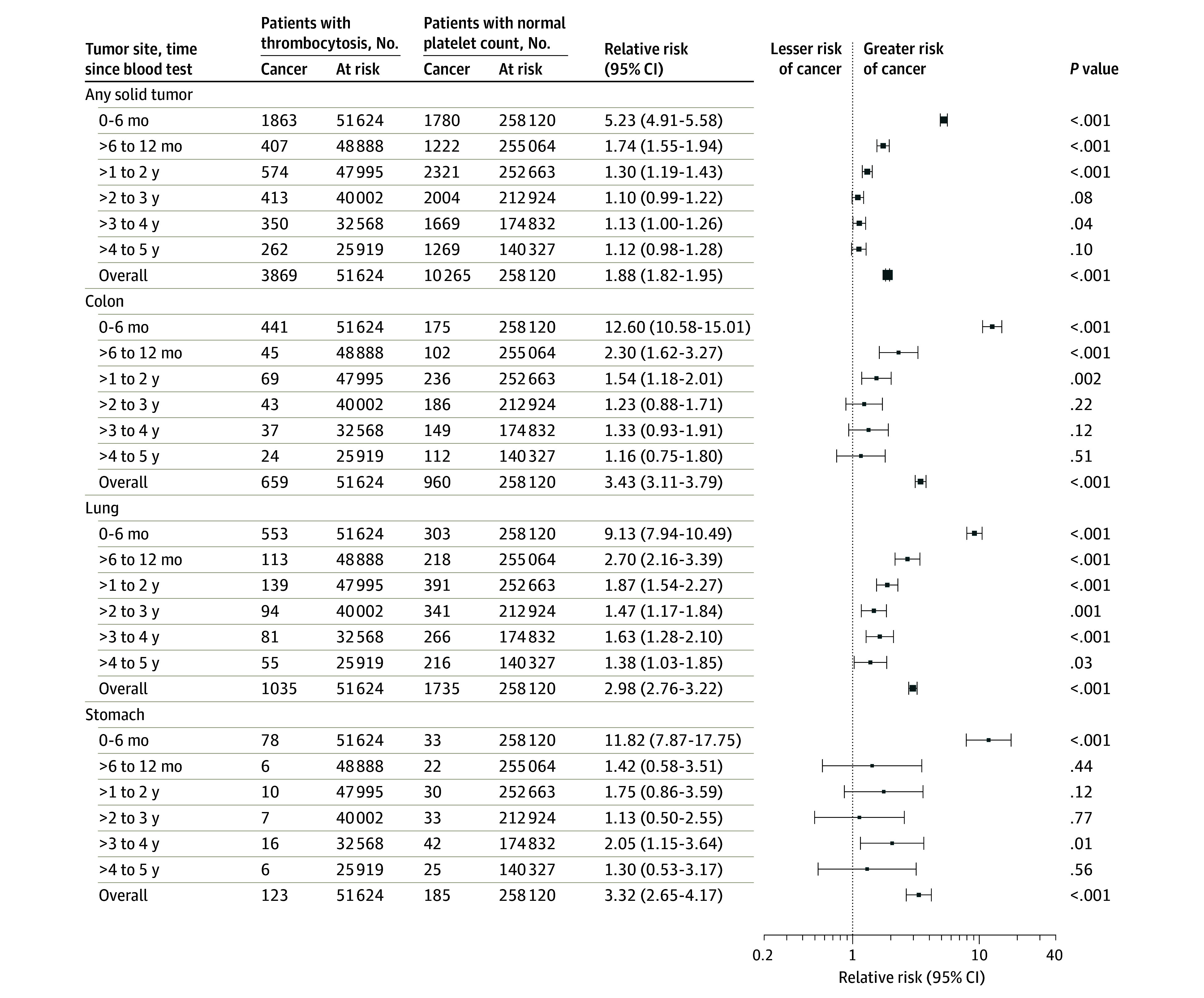
Relative Risks for the Development of Incident Cancer Among Individuals With Thrombocytosis Compared With Control Individuals With a Normal Platelet Count by Time Elapsed Since Blood Test and Cancer Site Data markers represent relative risks, with horizontal lines representing 95% CIs; marker size reflects the number of observed outcomes.

**Figure 3.**
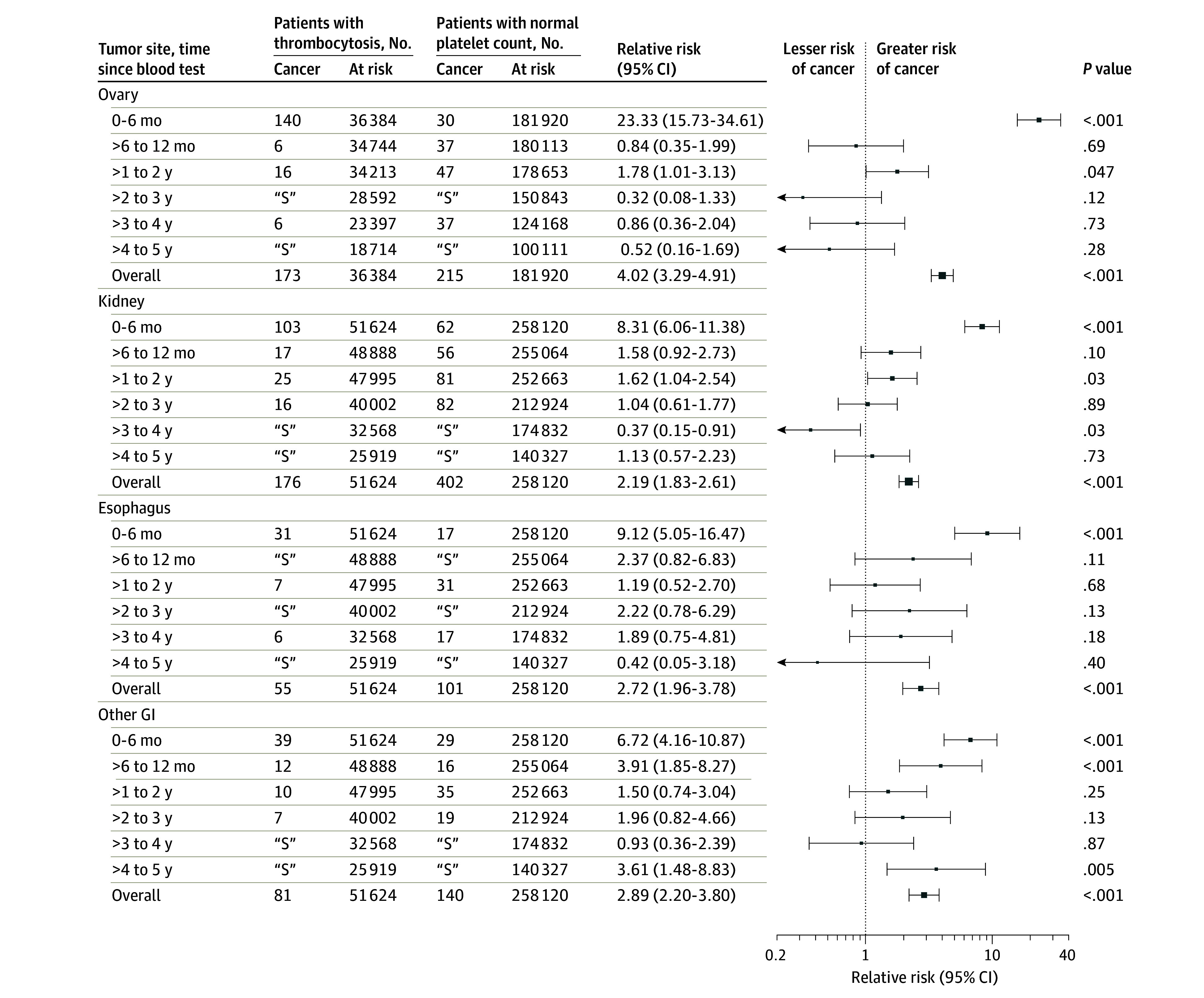
Relative Risks for the Development of Incident Cancer Among Individuals With Thrombocytosis Compared With Control Individuals With a Normal Platelet Count by Time Elapsed Since Blood Test and Cancer Site Data markers represent relative risks, with horizontal lines representing 95% CIs; marker size reflects the number of observed outcomes. GI indicates gastrointestinal. “S” indicates data were suppressed to avoid potential identification of individuals when 5 or fewer values were obtained.

**Figure 4.**
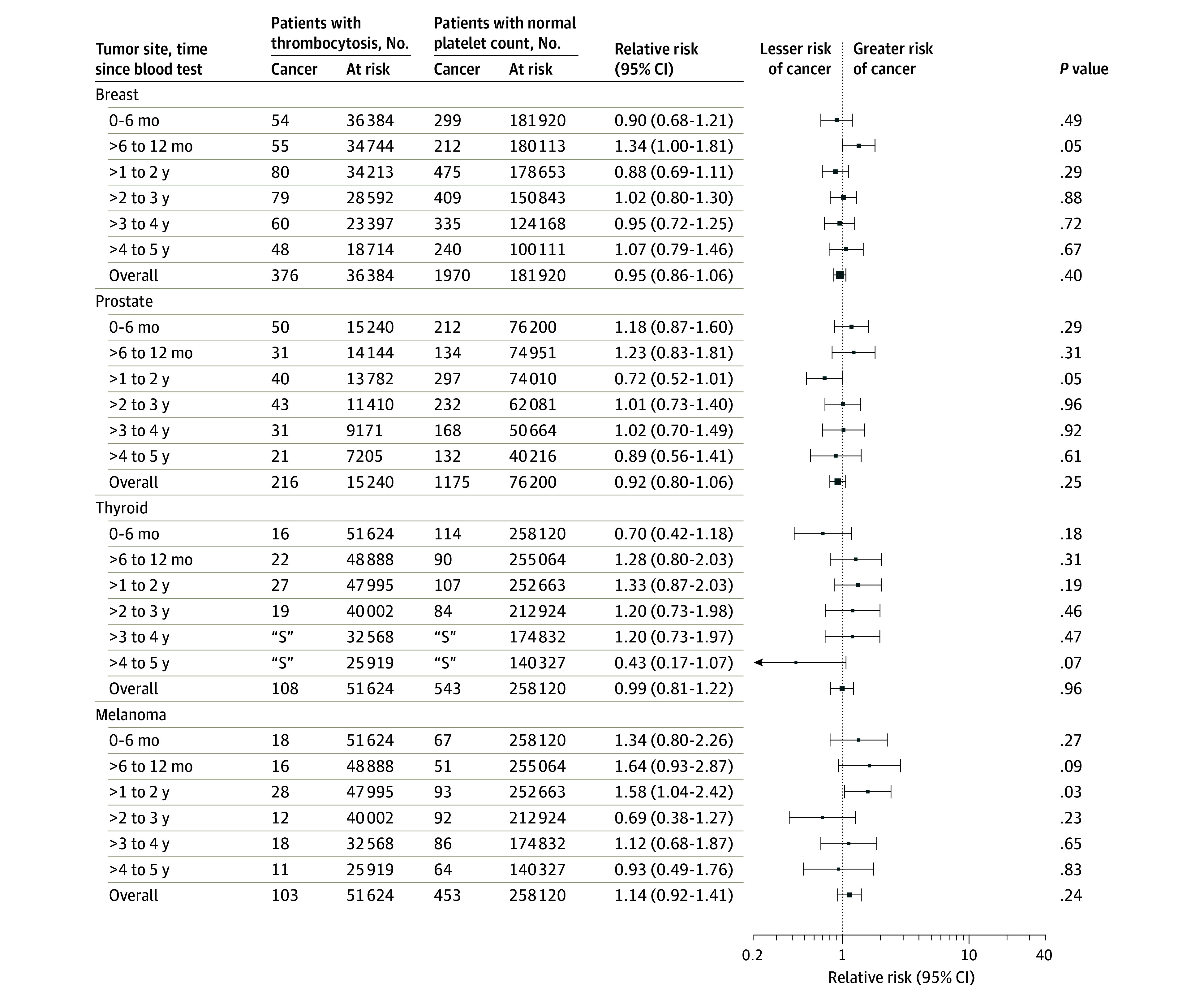
Relative Risks for the Development of Incident Cancer Among Individuals With Thrombocytosis Compared With Control Individuals With a Normal Platelet Count by Time Elapsed Since Blood Test and Cancer Site Data markers represent relative risks, with horizontal lines representing 95% CIs; marker size reflects the number of observed outcomes.

The relative risks by time elapsed from the index date for 15 solid-tumor sites are presented in Figure 2, Figure 3, and Figure 4, and these data for additional cancer sites are given in eFigures 3-5 in the Supplement. The cancers most commonly diagnosed within 2 years of the index CBC test are shown in the Table. The 2-year risk by each subgroup and by cancer type is provided in eTable 6 in the Supplement. The short-term (0-6-month) relative risk of incident cancer by cancer site is provided in eTable 9 in the Supplement. After restricting CBC tests to those ordered by an individual’s family doctor (eTable 8 in the Supplement), the 2-year relative risk of a solid-tumor diagnosis was 2.71 (95% CI, 2.57-2.87). In the sensitivity analysis involving a lower exposure cutoff value (400 × 109 platelets/L), the 2-year relative risk for a solid tumor diagnosis was 2.37 (95% CI, 2.29-2.45) (eTable 8 in the Supplement).

## Discussion

In this retrospective cohort study, we found a statistically significant association between a high platelet count and the development of a solid tumor within 5 years of the detection of thrombocytosis. The relative risks were most pronounced for lung cancer, ovarian cancer, kidney cancer, and gastrointestinal malignant tumors (esophageal cancer, stomach cancer, colorectal cancer, and other gastrointestinal malignant tumors).

The relative risk was greatest during the 6-month period after the diagnosis of thrombocytosis and decreased rapidly thereafter. This finding suggests that thrombocytosis may be a clinical marker for the presence of an existing cancer rather than being a factor associated with increased cancer risk and that cancer may lead to thrombocytosis rather than thrombocytosis being associated with the risk of developing a new cancer. If thrombocytosis is associated with cancer, instead of being a marker of cancer, the risk period would likely be more prolonged. In this study, for cancers associated with an increased short-term mortality rate, such as pancreatic and esophageal cancer, the risk of cancer after a diagnosis of thrombocytosis decreased rapidly. The risk of pancreatic cancer was 3.8-fold higher than that expected during the first 6 months after a diagnosis of thrombocytosis but was 1.3-fold higher from 6 months to 5 years after the diagnosis. The risk of esophageal cancer was 9.1-fold higher than expected during the first 6 months after a diagnosis of thrombocytosis but was 1.5-fold higher from 6 months to 5 years after diagnosis. An increased risk of cancer after a diagnosis of thrombocytosis was not seen for all cancer sites. Of note, there was no increased risk associated with breast cancer, prostate cancer, or thyroid cancer. In this study, for ovarian cancer, the short-term relative risk was very high (23.33; 95% CI, 15.73-34.61), and 0.4% of female patients with thrombocytosis received a diagnosis of ovarian cancer within 2 years. For these patients, the absolute risk of ovarian cancer was similar to that of breast cancer in the first 2 years after a diagnosis of thrombocytosis. Previous studies have also described an association between a high platelet count and the subsequent risk of cancer, including a series of studies from the UK.^10,11,12,14^

The association between platelet count and cancer is complex and involves inflammation, wound healing, angiogenesis, and the maintenance of vascular integrity.^6,7,8^ Platelet granules contain many cytokines, angiogenic factors, bioactive lipids, and mediators of angiogenesis that are released upon platelet activation.^6^ Platelets are the largest repository of vascular endothelial growth factor in the blood.^15^

The specific mechanisms whereby platelet functions affect carcinogenesis are unclear. In principle, high platelet counts may occur because of increased platelet production or reduced platelet turnover. Given that the bone marrow produces approximately 100 billion platelets per day,^16^ the platelet count is kept within narrow limits. Stone and colleagues^9^ showed that some serous ovarian cancers promote platelet formation through the production of interleukin 6, which in turn leads to increased production of thrombopoietin by the liver. Thrombopoietin is the main regulator of platelet concentration in the blood,^17^ and it would be of interest to examine whether thrombopoietin levels are high in patients with high platelet counts and cancer at other sites.

Based on the cancer distribution during the first year of follow-up in this study’s cohort, 5.6% of men and 3.9% of women may have had an occult cancer when thrombocytosis was diagnosed. Screening may include colonoscopy or fecal immunochemical testing for colorectal cancer, cancer antigen 125 testing and/or transvaginal ultrasonography for ovarian cancer, and a chest x-ray and spiral computed tomography scan for lung cancer. Current recommendations for lung cancer screening include screening individuals with a risk of 1.5% for developing lung cancer in a 6-year period,^18^ and this threshold is met by both men and women who have thrombocytosis. Other factors, such as smoking history, are likely to be relevant. The current study found that a high platelet count was associated with a greater risk of cancer of the kidney and cervix during the first 6 months after diagnosis of thrombocytosis; however, the absolute risk of these cancers was small. It is not clear from this study’s data whether individuals with thrombocytosis and negative screening test results should be considered as patients at high risk of cancer beyond the first screening. In this study, the presence of thrombocytosis was associated with greater risk of hematologic cancer and may indicate that a thorough health evaluation is needed.

The primary analysis focused on thrombocytosis, using the definition of a platelet count greater than 450 × 10^9^/L. In a sensitivity analysis, we defined thrombocytosis as a platelet count greater than 400 × 10^9^/L. The findings were comparable with those of the primary analysis (eTable 7 in the Supplement). The 2-year relative risk for a solid-tumor diagnosis was 2.67 (95% CI, 2.56-2.79) in the primary analysis and 2.37 (95% CI, 2.29-2.45) in the sensitivity analysis. The findings were also similar after restricting CBC tests to those ordered by an individual’s family doctor (eTable 8 in the Supplement); the 2-year relative risk of a solid-tumor diagnosis was 2.71 (95% CI, 2.57-2.87).

Future studies of this cohort are planned to assess the risk of cancer across the full range of platelet levels and explore the association between a low platelet count and risk of cancer. Furthermore, we plan to investigate whether expanding the examination of information contained in the CBC test result other than platelet count will enable us to refine the risk estimates. We also plan to investigate the association of an increasing platelet count with subsequent risk of cancer. Best et al^19^ studied RNA fragments contained in platelets from patients with cancer (some of the fragments were derived from the cancer) and found associations between the specific cancer site and the RNA profile. Other blood-based tests being investigated include methylation signatures in cell-free DNA,^20^ and it may be efficient to combine the results of 1 or more blood-based tests for cancer. This may enable the identification of individuals at high risk of cancer before they achieve the level of standard thrombocytosis.

### Limitations

This study has limitations. The data suggest that the cancers were present at the time of detection of thrombocytosis. We were unable to distinguish CBC tests that may have been performed because of an indication for an occult cancer (eg, symptoms). In some cases, the symptoms of cancer may have prompted a visit to a family doctor and the CBC test was diagnostic. In other cases, the increased platelet count may have been an unexpected finding during a routine blood test. However, the relative risk for all solid tumors in the interval from 3 months to 1 year was 1.99 (95% CI, 1.83-2.17), and this suggests that not all cancers diagnosed in the first year were clinically suspected on the index date (eFigure 2 in the Supplement).

## Conclusions

This cohort study found that an increased platelet count among individuals with normal platelet levels in the prior 2 years was associated with an increased risk of cancer. Increased risk of a solid-tumor diagnosis was predominant among lung, ovarian, kidney, and gastrointestinal malignant tumors. The findings suggest that individuals with unexplained thrombocytosis should be offered screening for several common cancers.

## Notes

Supplement. **eTable 1.** Description of administrative data sets used **eTable 2.** Definitions of descriptive variables **eTable 3.** Definitions of site-specific cancer events **eTable 4.** Descriptive table of unmatched cohort **eTable 5.** Descriptive table of matched cohort **eTable 6.** Risk of having an incident cancer within 2 years of thrombocytosis or a normal platelet count (matched controls), primary analysis **eTable 7.** Risk of having an incident cancer within 2 years of thrombocytosis or a normal platelet count (matched controls), sensitivity analysis 1 **eTable 8.** Risk of having an incident cancer within 2 years of thrombocytosis or a normal platelet count (matched controls), sensitivity analysis 2 **eTable 9.** Relative risk of having an incident cancer within 5 years of thrombocytosis or a normal platelet count (matched controls) **eFigure 1.** Flow diagram of inclusion cohort and exclusion criteria **eFigure 2.** Cancer incidence rates by week of follow-up among thrombocytosis patients and matched controls (primary analysis) **eFigure 3.** Relative risks for the development of incident cancer for those with thrombocytosis compared with those with a normal platelet count, by time elapsed since blood test and by cancer site (cervix, endometrium, bladder, and pancreas) **eFigure 4.** Relative risks for the development of incident cancer for those with thrombocytosis compared with those with a normal platelet count, by time elapsed since blood test and by cancer site (brain, head and neck, other solid tumor, and any hematologic tumor) **eFigure 5.** Relative risks for the development of incident cancer for those with thrombocytosis compared with those with a normal platelet count, by time elapsed since blood test and by cancer site (leukemia, lymphoma, multiple myeloma, and other hematologic tumor)
eReferences
