# Supplementary material for: Incidence of Cancer Among Adults With Thrombocytosis in Ontario, Canada
Source: JAMA Netw Open. 2021 Aug 12;4(8):e2120633. doi: 10.1001/jamanetworkopen.2021.20633 (PMC12011340; doi:10.1001/jamanetworkopen.2021.20633)
Supplement: Supplement. — eTable 1. Description of administrative data sets used eTable 2. Definitions of descriptive variables eTable 3. Definitions of site-specific cancer events eTable 4. Descriptive table of unmatched cohort eTable 5. Descriptive table of matched cohort eTable 6. Risk of having an incident cancer within 2 years of thrombocytosis or a normal platelet count (matched controls), primary analysis eTable 7. Risk of having an incident cancer within 2 years of thrombocytosis or a normal platelet count (matched controls), sensitivity analysis 1 eTable 8. Risk of having an incident cancer within 2 years of thrombocytosis or a normal platelet count (matched controls), sensitivity analysis 2 eTable 9. Relative risk of having an incident cancer within 5 years of thrombocytosis or a normal platelet count (matched controls) eFigure 1. Flow diagram of inclusion cohort and exclusion criteria eFigure 2. Cancer incidence rates by week of follow-up among thrombocytosis patients and matched controls (primary analysis) eFigure 3. Relative risks for the development of incident cancer for those with thrombocytosis compared with those with a normal platelet count, by time elapsed since blood test and by cancer site (cervix, endometrium, bladder, and pancreas) eFigure 4. Relative risks for the development of incident cancer for those with thrombocytosis compared with those with a normal platelet count, by time elapsed since blood test and by cancer site (brain, head and neck, other solid tumor, and any hematologic tumor) eFigure 5. Relative risks for the development of incident cancer for those with thrombocytosis compared with those with a normal platelet count, by time elapsed since blood test and by cancer site (leukemia, lymphoma, multiple myeloma, and other hematologic tumor) eReferences [file jamanetwopen-e2120633-s001.pdf]

Supplementary Online Content

Giannakeas V, Narod SA. Incidence of cancer among adults with thrombocytosis in Ontario, Canada. *JAMA Netw Open*. 2021;4(8):e2120633.
doi:10.1001/jamanetworkopen.2021.20633

eTable 1. Description of administrative data sets used
eTable 2. Definitions of descriptive variables
eTable 3. Definitions of site-specific cancer events
eTable 4. Descriptive table of unmatched cohort
eTable 5. Descriptive table of matched cohort
eTable 6. Risk of having an incident cancer within 2 years of thrombocytosis or a normal platelet count (matched controls), primary analysis
eTable 7. Risk of having an incident cancer within 2 years of thrombocytosis or a normal platelet count (matched controls), sensitivity analysis 1
eTable 8. Risk of having an incident cancer within 2 years of thrombocytosis or a normal platelet count (matched controls), sensitivity analysis 2
eTable 9. Relative risk of having an incident cancer within 5 years of thrombocytosis or a normal platelet count (matched controls)
eFigure 1. Flow diagram of inclusion cohort and exclusion criteria
eFigure 2. Cancer incidence rates by week of follow-up among thrombocytosis patients and matched controls (primary analysis)
eFigure 3. Relative risks for the development of incident cancer for those with thrombocytosis compared with those with a normal platelet count, by time elapsed since blood test and by cancer site (cervix, endometrium, bladder, and pancreas)
eFigure 4. Relative risks for the development of incident cancer for those with thrombocytosis compared with those with a normal platelet count, by time elapsed since blood test and by cancer site (brain, head and neck, other solid tumor, and any hematologic tumor)
eFigure 5. Relative risks for the development of incident cancer for those with thrombocytosis compared with those with a normal platelet count, by time elapsed since blood test and by cancer site (leukemia, lymphoma, multiple myeloma, and other hematologic tumor)
eReferences

This supplementary material has been provided by the authors to give readers additional information about their work.

© 2021 Giannakeas V et al. *JAMA Network Open*.

eTable 1. Description of administrative datasets used

Datasets	Years used	Description of use
Population and demographics		
Client Agency Program Enrollment (CAPE)	1999 – 2017	The CAPE dataset indicates an Ontario resident's enrollment to a primary care/ family doctor along with a start and end date of the enrollment.<br><br>The CAPE dataset was used to determine what individuals in the cohort were rostered to a primary care physician, and to identify individuals whose primary care provider ordered the index CBC test.
Registered Persons Database (RPDB)	1991 – 2018	The RPDB dataset provides basic demographic information on all Ontario residents registered with provincial health coverage. Demographic variables in the RPDB include date of birth, death date, sex, and postal code (updated annually). Start and end dates of provincial health coverage enrollment are also provided.<br><br>The RPDB dataset was used to get an individual's age (using date of birth) and sex. Neighborhood income quintile and residence location were derived from postal code information. Start and end dates for provincial health coverage (OHIP) and death date were used to determine eligibility as well as censoring events in the follow-up period.
Health services		
Discharge Abstract Database (DAD)	2002 – 2017	The DAD dataset includes patient-level data for acute care hospitalizations. The data has clinical details for each hospitalization such as clinical diagnosis (ICD-10) and health-related interventions (CCI).<br><br>Data from the DAD was used to contribute to aggregated diagnosis groups[1] (ADGs) as well as chronic condition definitions.
Ontario Health Insurance Plan Claims (OHIP)	2002 – 2017	The OHIP dataset includes physician billings to the province of Ontario among residents with provincial health coverage. Each billing includes a data element on the physician ID, the physician specialty, the patient ID, a diagnostic code (ICD-9), a fee code, and the service date.

© 2021 Giannakeas V et al. *JAMA Network Open*.

Datasets	Years used	Description of use
		Data from the OHIP dataset was used to obtain variables for primary care visits, ADGs, comorbidities, and chronic conditions.
Ontario Laboratories Information System (OLIS)	2007 – 2017	The OLIS dataset includes laboratory test results among tests being conducted in Ontario.<br><br>The data was used to acquire a dataset of complete blood count (CBC) tests among Ontario residents in the accrual period. These CBC tests were incorporated to define cohort entry, exclusions based on CBC values, and index date(s) among eligible subjects.
Same Day Surgery (SDS)	2002 – 2017	The SDS dataset contains patient-level information on same day surgeries, with a similar format to the DAD data.<br><br>Data from the SDS data was used to assign ADGs as well as chronic conditions.
Registries		
Ontario Cancer Registry (OCR)	1964 – 2018	The OCR dataset includes all reportable incident cancer diagnosis in Ontario between January 1964 and September 2019. Each incident cancer diagnosis has information on the cancer site/topology (ICD-10) and cancer morphology (ICD-O-3).<br><br>The OCR data was used to identify individuals with a history of cancer diagnosed in Ontario (exclusion). The data was also used to acquire an outcome event; any first-primary incident cancer diagnosis in the 5-year follow-up period.
ICES derived cohorts		
Ontario Asthma Dataset (ASTHMA)	1993 – 2017	A population-level cohort of Ontario residents with asthma.<br><br>A validated algorithm using DAD and OHIP data[2]:<br>Sensitivity = 80.6%<br>Specificity = 81.4%<br>PPV = 72.5%<br>NVP = 87.3%
Congestive Heart Failure (CHF)	1991 – 2017	A population-level cohort of Ontario residents with congestive heart failure.<br><br>A validated algorithm using DAD, NACRS and OHIP data[3]:<br>Sensitivity = 84.8%<br>Specificity = 97.0%<br>PPV = 55.6%

© 2021 Giannakeas V et al. *JAMA Network Open*.

Datasets	Years used	Description of use
Chronic Obstructive Pulmonary Disease (COPD)	1991 – 2017	A population-level cohort of Ontario residents with chronic obstructive pulmonary disease.<br><br>Validated algorithm using DAD and OHIP data[4]:<br>Sensitivity = 85.0%<br>Specificity = 78.4%
Ontario Dementia Database (DEMENTIA)	1996 – 2017	A population-level cohort of Ontario residents with dementia.<br><br>Validated algorithm using DAD, SDS, ODB and OHIP data[5]:<br>Sensitivity = 79.3%<br>Specificity = 99.1%
Ontario HIV Database (HIV)	1991 – 2017	A population-level cohort of Ontario residents with HIV.<br><br>Validated algorithm using OHIP data[6]:<br>Sensitivity = 96.2%<br>Specificity = 99.6%
Ontario Hypertension dataset (HYPER)	1991 – 2017	A population-level cohort of Ontario residents with hypertension.<br><br>Validated algorithm using DAD and OHIP data[7]:<br>Sensitivity = 72%<br>Specificity = 95%<br>PPV = 87%<br>NVP = 88%
Ontario Crohn's and Colitis Cohort dataset (OCCC)	1991 – 2017	A population-level cohort of Ontario residents with Crohn's and Colitis.<br><br>Validated algorithm using DAD, NACRS and OHIP data[8]:<br>Sensitivity = 59.3%<br>Specificity = 99.0%<br>PPV = 71.1%<br>NVP = 98.3%
Ontario Diabetes Dataset (ODD)	1991 – 2017	A population-level cohort of Ontario residents with diabetes.<br><br>Validated algorithm using DAD and ODB data[9]:<br>Sensitivity = 90.0%<br>Specificity = 97.7%<br>PPV = 92.6%
Ontario Rheumatoid Arthritis Dataset (ORAD)	1993 – 2017	A population-level cohort of Ontario residents with rheumatoid arthritis.<br><br>Validated algorithm using DAD and OHIP data[10]:<br>Sensitivity = 78%<br>Specificity = 100%<br>PPV = 78%<br>NVP = 100%

ICD = International Classification of Disease; CCI = Canadian Classification of Health Interventions; PPV = Positive Predictive Value; NPV = Negative Predictive Value; ODB = Ontario Drug Benefits Dataset

eTable 2. Definitions of descriptive variables

Variable Class	Definition	Data source(s)	Code type	Codes	Conditions/Notes
Primary care services utilization	Core primary care visit	OHIP	Fee code	A001 - MINOR ASSESS.-F.P./G.P.<br>A002 - Family Practice & Practice in General - Enhanced 18 month well baby visit<br>A003 - GEN. ASSESS. -F.P./G.P.<br>A004 - GEN.RE-ASSESS-F.P./G.P.<br>A005 - CONSULTATION -F.P./G.P.<br>A007 - INTERMED.ASSESS/WELL BABY CARE-F.P./G.P./PAED.<br>A008 - MINI ASSESSMENT-F.P./G.P.<br>A903 - GEN/FAM PRACT-PRE-DENTAL/OPER.ASSESS LIMIT 2 PER YEAR/PT<br>E075 - GERIATRIC GENERAL ASSESSMENT PREMIUM<br>G212 - D./T. PROC.-ALLERGY-HYPOSENSITIZATION INJECTION PLUS BASIC<br>G271 - D./T. PROC.-CARDIOV.-ANTICOAGULANT SUPERVISION<br>G372 - D./T. PROC.-INJECTIONS-INTRADERMAL/MUSCULAR ETC. EA. ADD.<br>G373 - D./T. PROC.-INJ. INTRADERMAL/MUSC. BASIC FEE (SHICK TEST)<br>G365 - D./T. PROC.-GYNAECOLOGY-PAPANICOLAOU SMEAR<br>G538 - D&T IMMUNIZATION-WITH VISIT, EACH INJECT.<br>G539 - Injection of unspecified agent - sole reason (first injection)<br>G590 - INFLUENZA AGENT +VISIT<br>G591 - Injection of influenza agent - sole reason<br>K005 - INDIVIDUAL CARE PER 1/2 HR<br>K013 - COUNSELLING-ONE OR MORE PEOPLE-PER 1/2HR.<br>K017 - ANNUAL HEALTH EXAM-CHILD AFT. 2ND BIRTHDAY.<br>P004 - OBS.-PRENATAL CARE-MINOR PRENATAL ASSESS.-SUBSEQ.PRENAT.VIS.<br>K130 - Periodic health visit - adolescent<br>K131 - Periodic health visit - adult aged 18 to 64 inclusive<br>K132 - Periodic health visit - adult 65 years of age and older<br>K030 - DIABETIC MANAGEMENT FEE	Note: All core primary care visits were restricted to OHIP billings from primary care providers (GP and community medicine specialties). A maximum of one core visit per patient-day-physician was counted.

© 2021 Giannakeas V et al. *JAMA Network Open*.

Variable Class	Definition	Data source(s)	Code type	Codes	Conditions/Notes
Chronic conditions*	Osteoarthritis	OHIP<br>DAD/SDS	ICD-9<br>ICD-10	715, 274, 710, 711, 716, 718, 720, 727, 728, 729, 739<br>M15, M16, M17, M18, M19<br>M00, M01, M02, M03, M07, M10, M11, M12, M13, M14, M20, M21, M22, M23, M24, M25, M30, M31, M32, M33, M34, M35, M36, M65, M66, M67, M68, M69, M7	
	Cardiac Arrhythmia	OHIP<br>DAD/SDS	ICD-9<br>ICD-10	427<br>I480, I481	
	Mood disorders	OHIP<br>DAD/SDS	ICD-9<br>ICD-10	296, 300, 309, 311<br>F30, F31, F32, F33, F341, F348, F349, F38, F39, F40, F41, F42, F431, F432, F438, F44, F450, F451, F452, F48, F530, F680, F930, F99	
	Other mental health disorders	OHIP<br>DAD/SDS	ICD-9<br>ICD-10	291, 292, 295, 297, 298, 299, 301, 302, 303, 304, 305, 306, 307, 313, 314, 315, 319<br>F1, F2, F04, F050, F058, F059, F060, F061, F062, F063, F064, F07, F08, F340, F35, F36, F37, F430, F439, F453, F454, F458, F46, F47, F49, F50, F51, F52, F531, F538, F539, F54, F55, F56, F57, F58, F59, F60, F61, F62, F63, F64, F65, F66, F67, F681, F688, F69, F7, F8, F90, F91, F92, F931, F932, F933, F938, F393, F94, F95, F96, F97, F98	
	Osteoporosis	OHIP<br>DAD/SDS	ICD-9<br>ICD-10	733<br>M81, M82	
	Renal disease	OHIP<br>DAD/SDS	ICD-9<br>ICD-10	403, 404, 584, 585, 586, 451<br>N17, N18, N19, T824, Z492, Z992	
	Stroke	OHIP<br>DAD/SDS	ICD-9<br>ICD-10	430, 431, 432, 434, 436<br>I60, I61, I62, I63, I64	
	Chronic coronary disease	OHIP<br>DAD/SDS	ICD-9<br>ICD-10	411, 412, 413, 414<br>I20, I22, I23, I24, I25	
	Acute myocardial infarction	DAD/SDS	ICD-10	I21	

*****All chronic conditions were obtained from validated diagnostic codes[11]. Definitions were defined based on either: 1) minimum of 3 OHIP claims, each claim greater than 30 days apart and less than 2 years apart, or 2) minimum of one inpatient hospitalization with any of the diagnostic codes.

© 2021 Giannakeas V et al. *JAMA Network Open*.

eTable 3. Definitions of site-specific cancer events

Definition	Sub-definition	Data source(s)	Code type	Codes	Conditions/Notes
Incident cancer diagnosis	Any malignant neoplasm	OCR	ICD-10	C00-C97	
Malignant solid tumour*	Any primary solid tumour	OCR	ICD-10	C00-C97	
	Colon	OCR	ICD-10	C18 – Colon<br>C19 – Rectosigmoid junction<br>C20 – Rectum	
	Lung	OCR	ICD-10	C34 – Bronchus or lung	
	Breast	OCR	ICD-10	C50 – Breast	Female sex
	Ovary	OCR	ICD-10	C56 – Ovary<br>C570 – Fallopian tube	Female sex
	Cervical	OCR	ICD-10	C53 – Cervix	Female sex
	Endometrium	OCR	ICD-10	C54 – Corpus uteri	Female sex
	Prostate	OCR	ICD-10	C61 – Prostate	Male sex
	Thyroid	OCR	ICD-10	C73 – Thyroid gland	
	Pancreas	OCR	ICD-10	C25 – Pancreas	
	Stomach	OCR	ICD-10	C16 – Stomach	
	Kidney	OCR	ICD-10	C64 – Kidney<br>C65 – Renal Pelvis	
	Bladder	OCR	ICD-10	C67 – Bladder	
	Liver	OCR	ICD-10	C22 – Liver and intrahepatic bile ducts	
	Esophagus	OCR	ICD-10	C15 – Esophagus	
	Other gastrointestinal tract	OCR	ICD-10	C17 – Small intestine<br>C21 – Anus and anal canal<br>C23 – Gallbladder	
	Brain	OCR	ICD-10	C71 – Brain	
	Melanoma	OCR	ICD-10<br><br>ICD-O-3	C00-C97, **and one of**<br><br>8720/3 – Malignant Melanoma, NOS<br>8743/3 – Superficial Spreading Melanoma<br>8721/3 – Nodular Melanoma<br>8742/3 – Lentigo Maligna Melanoma<br>8744/3 – Acral Lentiginous Melanoma<br>8772/3 – Spindle Cell Melanoma, NOS<br>8745/3 – Desmoplastic Melanoma<br>8770/3 – Mixed Epithelioid and Spindle Cell Melanoma<br>8771/3 – Epithelioid Cell Melanoma<br>8730/3 – Amelanotic Melanoma<br>8723/3 – Malignant Melanoma, Regressing<br>8774/3 – Spindle Cell Melanoma, Type B	 <br>Note: All ICD-O-3 melanoma codes were derived from the cancer registry and are in order from most frequent to least frequent among melanoma pathologies.

© 2021 Giannakeas V et al. *JAMA Network Open*.

Definition	Sub-definition	Data source(s)	Code type	Codes	Conditions/Notes
				8761/3 – Malignant Melanoma in a Giant Pigmented Nevus<br>8740/3 – Malignant Melanoma in Junctional Nevus<br>8722/3 – Balloon Cell Melanoma<br>8773/3 – Spindle Cell Melanoma, type A<br>8746/3 – Mucosal Lentiginous Melanoma<br>8741/3 – Malignant Melanoma in Precancerous Melanosis	
	Head and neck	OCR	ICD-10	C00-C09 – Lip, oral cavity<br>C10 – Oropharynx<br>C11 – Nasopharynx<br>C13 – Hypopharynx<br>C12 – Piriform sinus<br>C14 – Lip, oral cavity and pharynx (ill-defined)<br>C30 – Nasal cavity and middle ear<br>C31 – Accessory sinuses<br>C32 – Larynx	
	Other solid tumour	OCR	ICD-10	C00-C97	Cancer event met solid tumour definition, but none of the site-specific definitions described above.
Malignant hematological, lymphoid and related tissue	Any hematologic tumour	OCR	ICD-O-3	**All lymphoma, multiple myeloma, leukemia, and other hematological tumour morphology listed below.**	
	Lymphoma	OCR	ICD-O-3	9590/3 - Malignant lymphoma, NOS<br>9591/3 - Non-Hodgkin lymphoma, NOS<br>9596/3 - B-cell lymphoma, unclassifiable, with features intermediate between diffuse large B-cell lymphoma and classic Hodgkin lymphoma<br>9597/3 - Primary cutaneous follicle centre lymphoma<br>9650/3 - Classic Hodgkin lymphoma<br>9651/3 - Lymphocyte-rich classic Hodgkin lymphoma<br>9652/3 - Mixed cellularity classic Hodgkin lymphoma<br>9653/3 - Lymphocyte-depleted classic Hodgkin lymphoma<br>9654/3 - Hodgkin lymphoma, lymphocyte depletion, diffuse fibrosis<br>9655/3 - Hodgkin lymphoma, lymphocyte depletion, reticular<br>9659/3 - Nodular lymphocyte predominant Hodgkin lymphoma<br>9661/3 - Hodgkin granuloma	

© 2021 Giannakeas V et al. *JAMA Network Open*.

Definition	Sub-definition	Data source(s)	Code type	Codes	Conditions/Notes
				9662/3 - Hodgkin sarcoma<br>9663/3 - Nodular sclerosis classic Hodgkin lymphoma<br>9664/3 - Hodgkin lymphoma, nodular sclerosis, cellular phase<br>9665/3 - Hodgkin lymphoma, nodular sclerosis, grade 1<br>9667/3 - Hodgkin lymphoma, nodular sclerosis, grade 2<br>9670/3 - Malignant lymphoma, small B lymphocytic, NOS<br>9671/3 - Lymphoplasmacytic lymphoma<br>9673/3 - Mantle cell lymphoma<br>9675/3 - Malignant lymphoma, mixed small and large cell, diffuse<br>9678/3 - Primary effusion lymphoma<br>9679/3 - Primary mediastinal (thymic) large B-cell lymphoma<br>9680/3 - Diffuse large B-cell lymphoma, NOS<br>9684/3 - Malignant lymphoma, large B-cell, diffuse, immunoblastic, NOS<br>9687/3 - Burkitt lymphoma<br>9688/3 - T-cell/histiocyte-rich large B-cell lymphoma<br>9689/3 - Splenic marginal zone lymphoma<br>9690/3 - Follicular lymphoma<br>9691/3 - Follicular lymphoma, grade 2<br>9695/3 - Follicular lymphoma, grade 1<br>9698/3 - Follicular lymphoma, grade 3<br>9699/3 - Extranodal marginal zone lymphoma of mucosa-associated lymphoid tissue (MALT lymphoma)<br>9700/3 - Mycosis fungoides<br>9701/3 - Sezary syndrome<br>9702/3 - Peripheral T-cell lymphoma, NOS<br>9705/3 - Angioimmunoblastic T-cell lymphoma<br>9708/3 - Subcutaneous panniculitis-like T-cell lymphoma<br>9709/3 - Primary cutaneous T-cell lymphoma<br>9712/3 - Intravascular large B-cell lymphoma<br>9714/3 - Anaplastic large cell lymphoma, ALK-positive<br>9716/3 - Hepatosplenic T-cell lymphoma<br>9717/3 - Enteropathy-associated T-cell lymphoma<br>9718/3 - Primary cutaneous anaplastic large cell lymphoma<br>9719/3 - Extranodal NK-/T-cell lymphoma, nasal type<br>9724/3 - Systemic EBV-positive T-cell lymphoma of childhood<br>9725/3 - Hydroa vacciniforme-like lymphoma	

© 2021 Giannakeas V et al. *JAMA Network Open*.

Definition	Sub-definition	Data source(s)	Code type	Codes	Conditions/Notes
				9726/3 - Primary cutaneous gamma-delta T-cell lymphoma<br>9728/3 - Precursor B-cell lymphoblastic lymphoma<br>9729/3 - Precursor T-cell lymphoblastic lymphoma, NOS<br>9735/3 - Plasmablastic lymphoma<br>9737/3 - ALK-positive large B-cell lymphoma<br>9738/3 - HHV8-positive diffuse large B-cell lymphoma, NOS	
	Multiple myeloma	OCR	ICD-O-3	9731/3 - Solitary plasmacytoma of bone<br>9732/3 - Plasma cell myeloma<br>9733/3 - Plasma cell leukemia<br>9734/3 - Extraosseous plasmacytoma	
	Leukemia	OCR	ICD-O-3	9742/3 - Mast cell leukemia<br>9800/3 - Leukemia, NOS<br>9801/3 - Acute undifferentiated leukemia<br>9805/3 - Acute biphenotypic leukemia<br>9806/3 - Mixed-phenotype acute leukemia with t(9;22)(q34.1;q11.2); BCR-ABL1<br>9807/3 - Mixed-phenotype acute leukemia with t(v;11q23.3); KMT2A-rearranged<br>9808/3 - Mixed-phenotype acute leukemia, B/myeloid, not otherwise specified<br>9809/3 - Mixed-phenotype acute leukemia, T/myeloid, not otherwise specified<br>9811/3 - B-lymphoblastic leukemia/lymphoma, NOS<br>9812/3 - B-lymphoblastic leukemia/lymphoma with t(9;22)(q34.1;q11.2); BCR-ABL1<br>9813/3 - B-lymphoblastic leukemia/lymphoma with t(v;11q23.3); KMT2A-rearranged<br>9814/3 - B-lymphoblastic leukemia/lymphoma with t(12;21) (p13.2;q22.1); ETV6-RUNX1<br>9815/3 - B-lymphoblastic leukemia/lymphoma with hyperdiploidy<br>9816/3 - B-lymphoblastic leukemia/lymphoma with hypodiploidy<br>9817/3 - B-lymphoblastic leukemia/lymphoma with t(5;14)(q31.1;q32.1); IGH/IL3<br>9818/3 - B-lymphoblastic leukemia/lymphoma with t(1;19)(q23;p13.3); TCF3-PBX1<br>9820/3 - Lymphoid leukemia, NOS<br>9823/3 - Chronic lymphocytic leukemia/small lymphocytic lymphoma<br>9826/3 - Burkitt cell leukemia	

© 2021 Giannakeas V et al. *JAMA Network Open*.

Definition	Sub-definition	Data source(s)	Code type	Codes	Conditions/Notes
				9827/3 - Adult T-cell leukemia/lymphoma<br>9831/3 - T-cell large granular lymphocytic leukemia<br>9832/3 - Prolymphocytic leukemia, NOS<br>9833/3 - B-cell prolymphocytic leukemia<br>9834/3 - T-cell prolymphocytic leukemia<br>9835/3 - Precursor cell lymphoblastic leukemia, NOS<br>9836/3 - Precursor B-cell lymphoblastic leukemia<br>9837/3 - T-lymphoblastic leukemia/lymphoma<br>9840/3 - Pure erythroid leukemia<br>9860/3 - Myeloid leukemia, NOS<br>9861/3 - Acute myeloid leukemia, NOS<br>9863/3 - Chronic myeloid leukemia, NOS<br>9865/3 - Acute myeloid leukemia with t(6;9)(p23;q34.1); DEK-NUP214<br>9866/3 - Acute promyelocytic leukemia with PML-RARA<br>9867/3 - Acute myelomonocytic leukemia<br>9869/3 - Acute myeloid leukemia with inv(3)(q21.3q26.2) or t(3;3)(q21.3;q26.2); GATA2, MECOM<br>9870/3 - Acute basophilic leukemia<br>9871/3 - Acute myeloid leukemia with inv(16)(p13.1q22) or t(16;16)(p13.1;q22); CBFB-MYH11<br>9872/3 - Acute myeloid leukemia with minimal differentiation<br>9873/3 - Acute myeloid leukemia without maturation<br>9874/3 - Acute myeloid leukemia with maturation<br>9875/3 - Chronic myeloid leukemia, BCR-ABL1-positive<br>9876/3 - Atypical chronic myeloid leukemia, BCR-ABL1-negative<br>9891/3 - Acute monoblastic and monocytic leukemia<br>9895/3 - Acute myeloid leukemia with myelodysplasia-related changes<br>9896/3 - Acute myeloid leukemia, t(8;21)(q22;q22.1); RUNX1-RUNX1T1<br>9897/3 - Acute myeloid leukemia with t(9;11)(p21.3;q23.3); KMT2A-MLLT3<br>9898/3 - Myeloid leukemia associated with Down Syndrome<br>9910/3 - Acute megakaryoblastic leukemia<br>9911/3 - Acute myeloid leukemia (megakaryoblastic) with t(1;22)(p13.3;q13.1); RBM15-MKL1<br>9940/3 - Hairy cell leukemia<br>9945/3 - Chronic myelomonocytic leukemia<br>9946/3 - Juvenile myelomonocytic leukemia	

© 2021 Giannakeas V et al. *JAMA Network Open*.

Definition	Sub-definition	Data source(s)	Code type	Codes	Conditions/Notes
				9948/3 - Aggressive NK-cell leukemia<br>9963/3 - Chronic neutrophilic leukemia<br>9964/3 - Chronic eosinophilic leukemia, NOS	
	Other hematological tumour			9727/3 - Blastic plasmacytoid dendritic cell neoplasm<br>9740/3 - Mast cell sarcoma<br>9741/3 - Systemic mastocytosis with an associated hematological neoplasm<br>9750/3 - Malignant histiocytosis<br>9751/3 - Langerhans cell histiocytosis, NOS<br>9754/3 - Langerhans cell histiocytosis, disseminated<br>9755/3 - Histiocytic sarcoma<br>9756/3 - Langerhans cell sarcoma<br>9757/3 - Indeterminate dendritic cell tumor<br>9758/3 - Follicular dendritic cell sarcoma<br>9759/3 - Fibroblastic reticular cell tumor<br>9760/3 - Immunoproliferative disease, NOS<br>9761/3 - Waldenstrom macroglobulinemia<br>9762/3 - Heavy chain diseases<br>9764/3 - Immunoproliferative small intestinal disease<br>9920/3 - Therapy-related myeloid neoplasms<br>9930/3 - Myeloid sarcoma<br>9931/3 - Acute panmyelosis with myelofibrosis<br>9950/3 - Polycythemia vera<br>9960/3 - Myeloproliferative neoplasm, NOS<br>9961/3 - Primary myelofibrosis<br>9962/3 - Essential thrombocythemia<br>9965/3 - Myeloid/lymphoid neoplasms with PDGFRA rearrangement<br>9966/3 - Myeloid/lymphoid neoplasms with PDGFRB rearrangement<br>9967/3 - Myeloid/lymphoid neoplasms with FGFR1 rearrangement<br>9971/3 - Polymorphic post-transplant lymphoproliferative disorders<br>9975/3 - Myelodysplastic/myeloproliferative neoplasm, unclassifiable<br>9980/3 - Myelodysplastic syndrome with single lineage dysplasia<br>9982/3 - Myelodysplastic syndrome with ring sideroblasts and single lineage dysplasia<br>9983/3 - Myelodysplastic syndrome with excess blasts<br>9984/3 - Refractory anemia with excess blasts in transformation	

© 2021 Giannakeas V et al. *JAMA Network Open*.

Definition	Sub-definition	Data source(s)	Code type	Codes	Conditions/Notes
				9985/3 - Myelodysplastic syndrome with multilineage dysplasia<br>9986/3 - Myelodysplastic syndrome with isolated del(5q)<br>9987/3 - Therapy-related myelodysplastic syndrome, NOS<br>9989/3 - Myelodysplastic syndrome, unclassifiable<br>9991/3 - Refractory neutropenia<br>9992/3 - Refractory thrombocytopenia	

**Solid tumours are morphologic codes that are not in hematological, lymphoid and related tissue definitions.

© 2021 Giannakeas V et al. *JAMA Network Open*.

eTable 4. Descriptive table of unmatched cohort. Each subject measured at first CBC observation with a normal platelet count and/or thrombocytosis.

Description	Value	Total	Thrombocytosis (Exposed)	Normal Platelet Count (Unexposed)	Standardized Difference
	Overall	3,426,821	53,339 (1.6%)	3,373,482 (98.4%)	
General demographics					
**Calendar year**	Mean (SD)	2012.5 (2.5)	2013.6 (2.4)	2012.5 (2.5)	0.47
	Median (IQR)	2012 (2010-2015)	2014 (2012-2016)	2012 (2010-2015)	
**Age**	Mean (SD)	55.5 (9.8)	58.8 (9.9)	55.5 (9.7)	0.34
	Median (IQR)	55.0 (47.3-63.4)	59.7 (50.2-67.4)	55.0 (47.3-63.3)	
	40-49	1,145,666 (33.4%)	12,995 (24.4%)	1,132,671 (33.6%)	0.2
	50-59	1,086,890 (31.7%)	14,170 (26.6%)	1,072,720 (31.8%)	0.12
	60-75	1,194,265 (34.9%)	26,174 (49.1%)	1,168,091 (34.6%)	0.3
**Sex**	Female	1,953,540 (57.0%)	37,349 (70.0%)	1,916,191 (56.8%)	0.28
	Male	1,473,281 (43.0%)	15,990 (30.0%)	1,457,291 (43.2%)	0.28
**Neighborhood income quintile**	1 - Low	586,230 (17.1%)	10,872 (20.4%)	575,358 (17.1%)	0.09
	2	653,859 (19.1%)	10,989 (20.6%)	642,870 (19.1%)	0.04
	3	690,304 (20.1%)	10,606 (19.9%)	679,698 (20.1%)	0.01
	4	743,764 (21.7%)	10,624 (19.9%)	733,140 (21.7%)	0.04
	5 - High	742,252 (21.7%)	10,049 (18.8%)	732,203 (21.7%)	0.07
	Missing	10,412 (0.3%)	199 (0.4%)	10,213 (0.3%)	
**Residence location**	Urban	3,100,409 (90.5%)	46,879 (87.9%)	3,053,530 (90.5%)	0.08
	Rural	323,596 (9.4%)	6,395 (12.0%)	317,201 (9.4%)	0.08
	Missing	2,816 (0.1%)	65 (0.1%)	2,751 (0.1%)	
**Time enrolled in provincial health coverage (years)**	Mean (SD)	19.3 (6.4)	21.3 (5.8)	19.2 (6.4)	0.35
	Median (IQR)	20.8 (18.5-23.4)	22.9 (20.1-25.2)	20.8 (18.4-23.4)	
Primary care services utilization					
**Core primary care visits two years prior to index date**	Any visits	3,370,494 (98.4%)	52,132 (97.7%)	3,318,362 (98.4%)	0.05
	Mean (SD)	3.2 (3.8)	3.6 (4.4)	3.2 (3.7)	0.1
	Median (IQR)	2 (1-4)	2 (1-5)	2 (1-4)	
**Patient rostered to family doctor**	Yes	2,922,300 (85.3%)	46,032 (86.3%)	2,876,268 (85.3%)	0.03
**Index CBC test ordered by family doctor, among rostered patients**	Yes	2,394,230 (81.9%)	34,434 (74.8%)	2,359,796 (82.0%)	0.18
Comorbidities and chronic conditions					
**Aggregate diagnosis groups**	Mean (SD)	6.4 (3.2)	8.4 (3.7)	6.4 (3.2)	0.59
	Median (IQR)	6 (4-8)	8 (6-11)	6 (4-8)	

© 2021 Giannakeas V et al. *JAMA Network Open*.

Description	Value	Total	Thrombocytosis (Exposed)	Normal Platelet Count (Unexposed)	Standardized Difference
	0 - 4 ADGs	1,064,074 (31.1%)	8,209 (15.4%)	1,055,865 (31.3%)	0.38
	5 - 9 ADGs	1,778,571 (51.9%)	25,330 (47.5%)	1,753,241 (52.0%)	0.09
	10+ ADGs	584,176 (17.0%)	19,800 (37.1%)	564,376 (16.7%)	0.47
**Chronic conditions**	Asthma	295,450 (8.6%)	7,223 (13.5%)	288,227 (8.5%)	0.16
	Congestive heart failure	66,584 (1.9%)	2,894 (5.4%)	63,690 (1.9%)	0.19
	Inflammatory bowel disease	16,190 (0.5%)	1,194 (2.2%)	14,996 (0.4%)	0.16
	Chronic obstructive pulmonary disease	97,100 (2.8%)	4,581 (8.6%)	92,519 (2.7%)	0.25
	HIV	6,032 (0.2%)	95 (0.2%)	5,937 (0.2%)	0
	Hypertension	1,307,818 (38.2%)	28,518 (53.5%)	1,279,300 (37.9%)	0.32
	Dementia	22,733 (0.7%)	1,092 (2.0%)	21,641 (0.6%)	0.12
	Diabetes	474,898 (13.9%)	12,487 (23.4%)	462,411 (13.7%)	0.25
	Chronic rheumatoid arthritis	57,130 (1.7%)	3,521 (6.6%)	53,609 (1.6%)	0.25
	Osteoarthritis	513,933 (15.0%)	14,149 (26.5%)	499,784 (14.8%)	0.29
	Mood disorder	526,708 (15.4%)	11,108 (20.8%)	515,600 (15.3%)	0.14
	Other mental health disorder	168,133 (4.9%)	4,953 (9.3%)	163,180 (4.8%)	0.17
	Osteoporosis	32,104 (0.9%)	820 (1.5%)	31,284 (0.9%)	0.06
	Renal disease	42,979 (1.3%)	2,761 (5.2%)	40,218 (1.2%)	0.23
	Stroke	26,453 (0.8%)	897 (1.7%)	25,556 (0.8%)	0.08
	Chronic coronary syndrome	162,614 (4.7%)	5,219 (9.8%)	157,395 (4.7%)	0.2
	Acute myocardial infarction	44,072 (1.3%)	1,734 (3.3%)	42,338 (1.3%)	0.13
Platelet counts					
**Platelet count (10^9/L)**	Mean (SD)	250.4 (63.4)	510.4 (74.2)	246.2 (54.0)	4.07
	Median (IQR)	241 (207-281)	485 (464-528)	240 (206-279)	
**Number of routine CBC tests in the two years prior**	Mean (SD)	1.5 (1.3)	3.3 (3.6)	1.5 (1.2)	0.65
	Median (IQR)	1 (1-2)	2 (1-4)	1 (1-2)	
Follow-up period					
**Follow-up time (years)**	Mean (SD)	4.2 (1.3)	3.5 (1.6)	4.2 (1.3)	0.45
	Median (IQR)	5.0 (3.5-5.0)	4.0 (2.2-5.0)	5.0 (3.5-5.0)	
**Incident cancer events**	Any cancer	140,962 (4.1%)	5,160 (9.7%)	135,802 (4.0%)	0.23
	Solid tumour	125,678 (3.7%)	3,980 (7.5%)	121,698 (3.6%)	0.17
	Hematologic tumour	15,284 (0.4%)	1,180 (2.2%)	14,104 (0.4%)	0.16

© 2021 Giannakeas V et al. *JAMA Network Open*.

eTable 5. Descriptive table of matched cohort.

Description	Value	Total	Thrombocytosis (Exposed)	Normal Platelet Count (Unexposed)	Standardized Difference
	Overall	309,744	51,624 (16.7%)	258,120 (83.3%)	
General demographics					
**Calendar year**	Mean (SD)	2013.7 (2.4)	2013.7 (2.4)	2013.7 (2.4)	0
	Median (IQR)	2014 (2012-2016)	2014 (2012-2016)	2014 (2012-2016)	
**Age**	Mean (SD)	58.8 (9.9)	58.8 (9.9)	58.8 (9.9)	0
	Median (IQR)	59.7 (50.2-67.4)	59.7 (50.2-67.4)	59.7 (50.2-67.4)	
	40-49	75,436 (24.4%)	12,574 (24.4%)	62,862 (24.4%)	0
	50-59	82,157 (26.5%)	13,694 (26.5%)	68,463 (26.5%)	0
	60-75	152,151 (49.1%)	25,356 (49.1%)	126,795 (49.1%)	0
**Sex**	Female	218,304 (70.5%)	36,384 (70.5%)	181,920 (70.5%)	0
	Male	91,440 (29.5%)	15,240 (29.5%)	76,200 (29.5%)	0
**Neighborhood income quintile**	1 - Low	61,420 (19.8%)	10,303 (20.0%)	51,117 (19.8%)	0
	2	63,374 (20.5%)	10,581 (20.5%)	52,793 (20.5%)	0
	3	61,999 (20.0%)	10,318 (20.0%)	51,681 (20.0%)	0
	4	62,649 (20.2%)	10,392 (20.1%)	52,257 (20.2%)	0
	5 - High	59,185 (19.1%)	9,848 (19.1%)	49,337 (19.1%)	0
	Missing	1,117 (0.4%)	182 (0.4%)	935 (0.4%)	
**Residence location**	Urban	272,908 (88.1%)	45,526 (88.2%)	227,382 (88.1%)	0
	Rural	36,466 (11.8%)	6,036 (11.7%)	30,430 (11.8%)	0
	Missing	370 (0.1%)	62 (0.1%)	308 (0.1%)	
**Time enrolled in provincial health coverage (years)**	Mean (SD)	21.3 (5.8)	21.3 (5.8)	21.2 (5.9)	0.02
	Median (IQR)	22.9 (20.0-25.1)	22.9 (20.1-25.2)	22.9 (20.0-25.1)	
Primary care services utilization					
**Core primary care visits two years prior to index date**	Any visits	304,365 (98.3%)	50,523 (97.9%)	253,842 (98.3%)	0.03
	Mean (SD)	3.8 (4.7)	3.5 (4.4)	3.8 (4.8)	0.06
	Median (IQR)	3 (1-5)	2 (1-5)	3 (1-5)	
**Patient rostered to family doctor**	Yes	268,795 (86.8%)	44,701 (86.6%)	224,094 (86.8%)	0.01
**Index CBC test ordered by family doctor, among rostered patients**	Yes	205,318 (76.4%)	33,543 (75.0%)	171,775 (76.7%)	0.04
Comorbidities and chronic conditions					
**Aggregate diagnosis groups**	Mean (SD)	8.2 (3.6)	8.2 (3.6)	8.2 (3.6)	0
	Median (IQR)	8 (6-11)	8 (6-11)	8 (6-11)	
	0 - 4 ADGs	49,288 (15.9%)	8,187 (15.9%)	41,101 (15.9%)	0
	5 - 9 ADGs	151,865 (49.0%)	25,242 (48.9%)	126,623 (49.1%)	0

© 2021 Giannakeas V et al. *JAMA Network Open*.

Description	Value	Total	Thrombocytosis<br><br>(Exposed)	Normal Platelet Count<br>(Unexposed)	Standardized Difference
	10+ ADGs	108,591 (35.1%)	18,195 (35.2%)	90,396 (35.0%)	0
**Chronic conditions**	Asthma	40,346 (13.0%)	6,796 (13.2%)	33,550 (13.0%)	0
	Congestive heart failure	14,364 (4.6%)	2,484 (4.8%)	11,880 (4.6%)	0.01
	Inflammatory bowel disease	3,514 (1.1%)	1,155 (2.2%)	2,359 (0.9%)	0.11
	Chronic obstructive pulmonary disease	22,744 (7.3%)	3,885 (7.5%)	18,859 (7.3%)	0.01
	HIV	544 (0.2%)	88 (0.2%)	456 (0.2%)	0
	Hypertension	163,643 (52.8%)	27,249 (52.8%)	136,394 (52.8%)	0
	Dementia	5,238 (1.7%)	879 (1.7%)	4,359 (1.7%)	0
	Diabetes	69,748 (22.5%)	11,771 (22.8%)	57,977 (22.5%)	0.01
	Chronic rheumatoid arthritis	16,532 (5.3%)	3,377 (6.5%)	13,155 (5.1%)	0.06
	Osteoarthritis	75,524 (24.4%)	13,494 (26.1%)	62,030 (24.0%)	0.05
	Mood disorder	63,256 (20.4%)	10,445 (20.2%)	52,811 (20.5%)	0.01
	Other mental health disorder	23,354 (7.5%)	4,321 (8.4%)	19,033 (7.4%)	0.04
	Osteoporosis	4,648 (1.5%)	774 (1.5%)	3,874 (1.5%)	0
	Renal disease	12,688 (4.1%)	2,223 (4.3%)	10,465 (4.1%)	0.01
	Stroke	5,083 (1.6%)	712 (1.4%)	4,371 (1.7%)	0.03
	Chronic coronary syndrome	27,791 (9.0%)	4,768 (9.2%)	23,023 (8.9%)	0.01
	Acute myocardial infarction	7,604 (2.5%)	1,503 (2.9%)	6,101 (2.4%)	0.03
Platelet counts					
**Platelet count (10^9/L)**	Mean (SD)<br>Median (IQR)	294.7 (112.5)<br>260 (218-326)	509.9 (73.7)<br>485 (464-528)	251.7 (54.7)<br>245 (211-285)	3.98
**Number of routine CBC tests in the two years prior**	Mean (SD)<br>Median (IQR)	3.0 (3.3)<br>2 (1-3)	3.2 (3.5)<br>2 (1-4)	3.0 (3.3)<br>2 (1-3)	0.07
Follow-up period					
**Follow-up time (years)**	Mean (SD)<br>Median (IQR)	3.7 (1.4)<br>4.3 (2.5-5.0)	3.5 (1.6)<br>4.0 (2.2-5.0)	3.7 (1.4)<br>4.3 (2.5-5.0)	0.15
**Incident cancer events**	Any cancer	16,687 (5.4%)	5,008 (9.7%)	11,679 (4.5%)	0.2
	Solid tumour	14,134 (4.6%)	3,869 (7.5%)	10,265 (4.0%)	0.15
	Hematologic tumour	2,553 (0.8%)	1,139 (2.2%)	1,414 (0.5%)	0.14

Note: Matched cohort includes individuals with thrombocytosis (exposed) matched to five individuals with a normal platelet count (unexposed) on age (rounded to nearest integer), sex, and propensity score. Propensity score incorporates demographic variables (residence location, living in long-term care, neighborhood income quintile), and comorbidities and selected chronic conditions (all ADGs[1], CHF[3], COPD[4], hypertension[7], diabetes[9]). We used a caliper of 0.2 times the standard deviation of the logit of the propensity score[12].

© 2021 Giannakeas V et al. *JAMA Network Open*.

eTable 6. Risk of having an incident cancer within two years of thrombocytosis or a normal platelet count (matched controls). Thrombocytosis defined as >450 platelets per μL (primary analysis).

	Thrombocytosis			Normal Platelet Count						
	N	***Events***	***Risk (per 1,000)***	***N***	***Events***	***Risk (per 1,000)***	**Relative Risk (95% CI)**	**P-Value**	**Risk Difference (per 1,000) (95% CI)**	**P-Value**
Any solid tumour										
Female	36,384	1,769	48.62	181,920	3,304	18.16	2.68 (2.53-2.83)	<.0001	30.5 ([28.2] - [32.8])	<.0001
Male	15,240	1,075	70.54	76,200	2,019	26.50	2.66 (2.48-2.86)	<.0001	44 ([39.8] - [48.3])	<.0001
Age 40 - 49	12,574	245	19.48	62,862	506	8.05	2.42 (2.08-2.82)	<.0001	11.4 ([8.9] - [14])	<.0001
Age 50 - 59	13,694	613	44.76	68,463	956	13.96	3.21 (2.9-3.54)	<.0001	30.8 ([27.2] - [34.4])	<.0001
Age 60 - 75	25,356	1,986	78.32	126,795	3,861	30.45	2.57 (2.44-2.71)	<.0001	47.9 ([44.4] - [51.3])	<.0001
**Any cancer**	51,624	3,593	69.60	258,120	6,019	23.32	2.98 (2.87-3.11)	<.0001	46.3 ([44] - [48.6])	<.0001
Any solid tumour	51,624	2,844	55.09	258,120	5,323	20.62	2.67 (2.56-2.79)	<.0001	34.5 ([32.4] - [36.5])	<.0001
Colon	51,624	555	10.75	258,120	513	1.99	5.41 (4.8-6.1)	<.0001	8.8 ([7.9] - [9.7])	<.0001
Lung	51,624	805	15.59	258,120	912	3.53	4.41 (4.02-4.85)	<.0001	12.1 ([11] - [13.2])	<.0001
Breast (female)	36,384	189	5.19	181,920	986	5.42	0.96 (0.82-1.12)	0.5918	-0.2 ([-1] - [0.6])	0.5865
Ovary (female)	36,384	162	4.45	181,920	114	0.63	7.11 (5.59-9.03)	<.0001	3.8 ([3.1] - [4.5])	<.0001
Cervix (female)	36,384	17	0.47	181,920	35	0.19	2.43 (1.36-4.33)	0.0027	0.3 ([0] - [0.5])	0.0197
Endometrium (female)	36,384	83	2.28	181,920	265	1.46	1.57 (1.22-2)	0.0004	0.8 ([0.3] - [1.3])	0.0019
Prostate (male)	15,240	121	7.94	76,200	643	8.44	0.94 (0.78-1.14)	0.5371	-0.5 ([-2.1] - [1.1])	0.5287
Thyroid	51,624	65	1.26	258,120	311	1.20	1.05 (0.8-1.37)	0.7466	0.1 ([-0.3] - [0.4])	0.7502
Pancreas	51,624	74	1.43	258,120	152	0.59	2.43 (1.84-3.21)	<.0001	0.8 ([0.5] - [1.2])	<.0001
Stomach	51,624	94	1.82	258,120	85	0.33	5.53 (4.12-7.41)	<.0001	1.5 ([1.1] - [1.9])	<.0001
Kidney	51,624	145	2.81	258,120	199	0.77	3.64 (2.94-4.51)	<.0001	2 ([1.6] - [2.5])	<.0001
Bladder	51,624	75	1.45	258,120	229	0.89	1.64 (1.26-2.13)	0.0002	0.6 ([0.2] - [0.9])	0.0014
Esophagus	51,624	43	0.83	258,120	59	0.23	3.64 (2.46-5.4)	<.0001	0.6 ([0.3] - [0.9])	<.0001
Other GI	51,624	61	1.18	258,120	80	0.31	3.81 (2.73-5.32)	<.0001	0.9 ([0.6] - [1.2])	<.0001
Brain	51,624	13	0.25	258,120	56	0.22	1.16 (0.63-2.12)	0.6283	0 ([-0.1] - [0.2])	0.6447
Melanoma	51,624	62	1.20	258,120	211	0.82	1.47 (1.11-1.95)	0.0077	0.4 ([0.1] - [0.7])	0.0182
Head and neck	51,624	43	0.83	258,120	163	0.63	1.32 (0.94-1.85)	0.1062	0.2 ([-0.1] - [0.5])	0.1393
Other solid tumour	51,624	199	3.85	258,120	258	1.00	3.86 (3.21-4.64)	<.0001	2.9 ([2.3] - [3.4])	<.0001
Any hematologic tumour	51,624	749	14.51	258,120	696	2.70	5.38 (4.86-5.96)	<.0001	11.8 ([10.8] - [12.9])	<.0001
Leukemia	51,624	28	0.54	258,120	37	0.14	3.78 (2.32-6.18)	<.0001	0.4 ([0.2] - [0.6])	<.0001
Lymphoma	51,624	237	4.59	258,120	405	1.57	2.93 (2.49-3.43)	<.0001	3 ([2.4] - [3.6])	<.0001
Multiple myeloma	51,624	50	0.97	258,120	150	0.58	1.67 (1.21-2.3)	0.0018	0.4 ([0.1] - [0.7])	0.0075
Other hematologic tumour	51,624	434	8.41	258,120	104	0.40	20.87 (16.85-25.84)	<.0001	8 ([7.2] - [8.8])	<.0001

eTable 7. Risk of having an incident cancer within two years of thrombocytosis or a normal platelet count (matched controls). Thrombocytosis defined as >400 platelets per μL (sensitivity analysis 1).

	Thrombocytosis	Normal Platelet Count	

© 2021 Giannakeas V et al. *JAMA Network Open*.

	N	*Events*	*Risk (per 1,000)*	*N*	*Events*	*Risk (per 1,000)*	**Relative Risk (95% CI)**	**P-Value**	**Risk Difference (per 1,000) (95% CI)**	**P-Value**
Any solid tumour										
Female	71,344	2,810	39.39	356,720	6,167	17.29	2.28 (2.18-2.38)	<.0001	22.1 ([20.6] - [23.6])	<.0001
Male	27,641	1,735	62.77	138,205	3,434	24.85	2.53 (2.39-2.67)	<.0001	37.9 ([34.9] - [40.9])	<.0001
Age 40 - 49	27,315	420	15.38	136,296	1,093	8.02	1.92 (1.71-2.14)	<.0001	7.4 ([5.8] - [8.9])	<.0001
Age 50 - 59	26,795	970	36.20	134,289	1,947	14.50	2.5 (2.31-2.69)	<.0001	21.7 ([19.4] - [24])	<.0001
Age 60 - 75	44,875	3,155	70.31	224,340	6,561	29.25	2.4 (2.31-2.51)	<.0001	41.1 ([38.6] - [43.5])	<.0001
**Any cancer**	98,985	5,421	54.77	494,925	10,789	21.80	2.51 (2.43-2.59)	<.0001	33 ([31.5] - [34.4])	<.0001
Any solid tumour	98,985	4,545	45.92	494,925	9,601	19.40	2.37 (2.29-2.45)	<.0001	26.5 ([25.2] - [27.9])	<.0001
Colon	98,985	856	8.65	494,925	925	1.87	4.63 (4.22-5.08)	<.0001	6.8 ([6.2] - [7.4])	<.0001
Lung	98,985	1,201	12.13	494,925	1,417	2.86	4.24 (3.93-4.58)	<.0001	9.3 ([8.6] - [10])	<.0001
Breast (female)	71,344	353	4.95	356,720	1,879	5.27	0.94 (0.84-1.05)	0.2794	-0.3 ([-0.9] - [0.2])	0.2693
Ovary (female)	71,344	236	3.31	356,720	188	0.53	6.28 (5.18-7.6)	<.0001	2.8 ([2.4] - [3.2])	<.0001
Cervix (female)	71,344	30	0.42	356,720	70	0.20	2.14 (1.4-3.29)	0.0005	0.2 ([0.1] - [0.4])	0.0052
Endometrium (female)	71,344	170	2.38	356,720	588	1.65	1.45 (1.22-1.71)	<.0001	0.7 ([0.4] - [1.1])	0.0002
Prostate (male)	27,641	213	7.71	138,205	1,055	7.63	1.01 (0.87-1.17)	0.8997	0.1 ([-1.1] - [1.2])	0.9000
Thyroid	98,985	113	1.14	494,925	618	1.25	0.91 (0.75-1.12)	0.3806	-0.1 ([-0.3] - [0.1])	0.3661
Pancreas	98,985	132	1.33	494,925	266	0.54	2.48 (2.01-3.06)	<.0001	0.8 ([0.6] - [1])	<.0001
Stomach	98,985	183	1.85	494,925	193	0.39	4.74 (3.87-5.8)	<.0001	1.5 ([1.2] - [1.7])	<.0001
Kidney	98,985	223	2.25	494,925	336	0.68	3.32 (2.8-3.93)	<.0001	1.6 ([1.3] - [1.9])	<.0001
Bladder	98,985	118	1.19	494,925	452	0.91	1.31 (1.07-1.6)	0.0099	0.3 ([0] - [0.5])	0.0179
Esophagus	98,985	69	0.70	494,925	107	0.22	3.22 (2.38-4.36)	<.0001	0.5 ([0.3] - [0.7])	<.0001
Other GI	98,985	86	0.87	494,925	140	0.28	3.07 (2.35-4.02)	<.0001	0.6 ([0.4] - [0.8])	<.0001
Brain	98,985	27	0.27	494,925	113	0.23	1.19 (0.79-1.82)	0.4062	0 ([-0.1] - [0.2])	0.4331
Melanoma	98,985	94	0.95	494,925	394	0.80	1.19 (0.95-1.49)	0.1242	0.2 ([-0.1] - [0.4])	0.1466
Head and neck	98,985	79	0.80	494,925	261	0.53	1.51 (1.18-1.95)	0.0012	0.3 ([0.1] - [0.5])	0.0046
Other solid tumour	98,985	297	3.00	494,925	488	0.99	3.04 (2.63-3.51)	<.0001	2 ([1.7] - [2.4])	<.0001
Any hematologic tumour	98,985	876	8.85	494,925	1,188	2.40	3.69 (3.38-4.02)	<.0001	6.4 ([5.9] - [7])	<.0001
Leukemia	98,985	37	0.37	494,925	70	0.14	2.64 (1.77-3.94)	<.0001	0.2 ([0.1] - [0.4])	0.0003
Lymphoma	98,985	372	3.76	494,925	720	1.45	2.58 (2.28-2.93)	<.0001	2.3 ([1.9] - [2.7])	<.0001
Multiple myeloma	98,985	90	0.91	494,925	227	0.46	1.98 (1.55-2.53)	<.0001	0.5 ([0.3] - [0.6])	<.0001
Other hematologic tumour	98,985	377	3.81	494,925	171	0.35	11.02 (9.2-13.21)	<.0001	3.5 ([3.1] - [3.9])	<.0001

© 2021 Giannakeas V et al. *JAMA Network Open*.

eTable 8. Risk of having an incident cancer within two years of thrombocytosis or a normal platelet count (matched controls). A subset of CBC observations ordered by an individual's family doctor (sensitivity analysis 2).

	Thrombocytosis			Normal Platelet Count						
	N	*Events*	*Risk (per 1,000)*	*N*	*Events*	*Risk (per 1,000)*	Relative Risk (95% CI)	P-Value	Risk Difference (per 1,000) (95% CI)	P-Value
Any solid tumour										
Female	23,504	1,202	51.14	117,520	2,199	18.71	2.73 (2.55-2.93)	<.0001	32.4 ([29.5] - [35.3])	<.0001
Male	9,508	690	72.57	47,540	1,286	27.05	2.68 (2.45-2.93)	<.0001	45.5 ([40.1] - [50.9])	<.0001
Age 40 - 49	7,490	138	18.42	37,386	311	8.32	2.21 (1.82-2.7)	<.0001	10.1 ([6.9] - [13.3])	<.0001
Age 50 - 59	8,535	395	46.28	42,682	666	15.60	2.97 (2.62-3.35)	<.0001	30.7 ([26.1] - [35.3])	<.0001
Age 60 - 75	16,987	1,359	80.00	84,992	2,508	29.51	2.71 (2.54-2.89)	<.0001	50.5 ([46.3] - [54.7])	<.0001
**Any cancer**	33,012	2,398	72.64	165,060	3,909	23.68	3.07 (2.92-3.22)	<.0001	49 ([46.1] - [51.9])	<.0001
Any solid tumour	33,012	1,892	57.31	165,060	3,485	21.11	2.71 (2.57-2.87)	<.0001	36.2 ([33.6] - [38.8])	<.0001
Colon	33,012	354	10.72	165,060	334	2.02	5.3 (4.57-6.15)	<.0001	8.7 ([7.6] - [9.8])	<.0001
Lung	33,012	570	17.27	165,060	487	2.95	5.85 (5.19-6.6)	<.0001	14.3 ([12.9] - [15.7])	<.0001
Breast (female)	23,504	131	5.57	117,520	718	6.11	0.91 (0.76-1.1)	0.3324	-0.5 ([-1.6] - [0.5])	0.3174
Ovary (female)	23,504	115	4.89	117,520	66	0.56	8.71 (6.44-11.79)	<.0001	4.3 ([3.4] - [5.2])	<.0001
Cervix (female)	23,504	10	0.43	117,520	16	0.14	3.13 (1.42-6.89)	0.0047	0.3 ([0] - [0.6])	0.0371
Endometrium (female)	23,504	54	2.30	117,520	191	1.63	1.41 (1.05-1.91)	0.0246	0.7 ([0] - [1.3])	0.0439
Prostate (male)	9,508	77	8.10	47,540	401	8.44	0.96 (0.75-1.22)	0.7425	-0.3 ([-2.3] - [1.6])	0.7390
Thyroid	33,012	36	1.09	165,060	193	1.17	0.93 (0.65-1.33)	0.7007	-0.1 ([-0.5] - [0.3])	0.6940
Pancreas	33,012	48	1.45	165,060	104	0.63	2.31 (1.64-3.25)	<.0001	0.8 ([0.4] - [1.3])	0.0002
Stomach	33,012	63	1.91	165,060	76	0.46	4.14 (2.97-5.79)	<.0001	1.4 ([1] - [1.9])	<.0001
Kidney	33,012	97	2.94	165,060	123	0.75	3.94 (3.02-5.14)	<.0001	2.2 ([1.6] - [2.8])	<.0001
Bladder	33,012	48	1.45	165,060	187	1.13	1.28 (0.93-1.76)	0.1228	0.3 ([-0.1] - [0.8])	0.1544
Esophagus	33,012	31	0.94	165,060	36	0.22	4.31 (2.66-6.96)	<.0001	0.7 ([0.4] - [1.1])	<.0001
Other GI	33,012	34	1.03	165,060	44	0.27	3.86 (2.47-6.04)	<.0001	0.8 ([0.4] - [1.1])	<.0001
Brain	33,012	6	0.18	165,060	34	0.21	0.88 (0.37-2.1)	0.7774	0 ([-0.2] - [0.1])	0.7681
Melanoma	33,012	44	1.33	165,060	165	1.00	1.33 (0.96-1.86)	0.0898	0.3 ([-0.1] - [0.8])	0.1218
Head and neck	33,012	29	0.88	165,060	99	0.60	1.46 (0.97-2.22)	0.0706	0.3 ([-0.1] - [0.6])	0.1089
Other solid tumour	33,012	123	3.73	165,060	172	1.04	3.58 (2.84-4.51)	<.0001	2.7 ([2] - [3.4])	<.0001
Any hematologic tumour	33,012	506	15.33	165,060	424	2.57	5.97 (5.25-6.79)	<.0001	12.8 ([11.4] - [14.1])	<.0001
Leukemia	33,012	17	0.51	165,060	31	0.19	2.74 (1.52-4.95)	0.0008	0.3 ([0.1] - [0.6])	0.0114
Lymphoma	33,012	160	4.85	165,060	266	1.61	3.01 (2.47-3.66)	<.0001	3.2 ([2.5] - [4])	<.0001
Multiple myeloma	33,012	27	0.82	165,060	72	0.44	1.88 (1.2-2.92)	0.0053	0.4 ([0.1] - [0.7])	0.0211
Other hematologic tumour	33,012	302	9.15	165,060	55	0.33	27.45 (20.6-36.58)	<.0001	8.8 ([7.8] - [9.8])	<.0001

eTable 9. Relative Risk of having an incident cancer within 5 years of thrombocytosis or a normal platelet count (matched controls). Thrombocytosis defined as >450 platelets per µL (primary analysis).

© 2021 Giannakeas V et al. *JAMA Network Open*.

Cancer Type	Overall (0 to 5 years)	0 to 6 months	>6 months to 5 years
**Any cancer**	2.14 (2.08 - 2.21)	5.54 (5.22 - 5.88)	1.50 (1.44 - 1.56)
Any solid tumour	1.88 (1.82 - 1.95)	5.23 (4.91 - 5.58)	1.23 (1.18 - 1.29)
Colon	3.43 (3.11 - 3.79)	12.60 (10.58 - 15.01)	1.45 (1.25 - 1.68)
Lung	2.98 (2.76 - 3.22)	9.13 (7.94 - 10.49)	1.76 (1.58 - 1.95)
Breast (female)	0.95 (0.86 - 1.06)	0.90 (0.68 - 1.21)	1.00 (0.89 - 1.12)
Ovary (female)	4.02 (3.29 - 4.91)	23.33 (15.73 - 34.61)	0.92 (0.64 - 1.34)
Cervix (female)	1.59 (0.96 - 2.62)	3.53 (1.69 - 7.39)	0.90 (0.43 - 1.91)
Endometrium (female)	1.25 (1.03 - 1.53)	2.73 (1.92 - 3.88)	0.98 (0.77 - 1.24)
Prostate (male)	0.92 (0.80 - 1.06)	1.18 (0.87 - 1.60)	0.91 (0.78 - 1.08)
Thyroid	0.99 (0.81 - 1.22)	0.70 (0.42 - 1.18)	1.12 (0.89 - 1.40)
Pancreas	1.81 (1.45 - 2.26)	3.75 (2.58 - 5.45)	1.34 (1.01 - 1.78)
Stomach	3.32 (2.65 - 4.17)	11.82 (7.87 - 17.75)	1.54 (1.11 - 2.15)
Kidney	2.19 (1.83 - 2.61)	8.31 (6.06 - 11.38)	1.12 (0.87 - 1.44)
Bladder	1.19 (0.97 - 1.45)	2.12 (1.43 - 3.13)	1.04 (0.82 - 1.31)
Esophagus	2.72 (1.96 - 3.78)	9.12 (5.05 - 16.47)	1.49 (0.95 - 2.35)
Other GI	2.89 (2.20 - 3.80)	6.72 (4.16 - 10.87)	1.97 (1.38 - 2.82)
Brain	0.92 (0.57 - 1.48)	1.14 (0.43 - 3.00)	0.90 (0.52 - 1.56)
Melanoma	1.14 (0.92 - 1.41)	1.34 (0.80 - 2.26)	1.15 (0.91 - 1.45)
Head and neck	1.21 (0.94 - 1.57)	1.52 (0.88 - 2.61)	1.19 (0.89 - 1.60)
Other solid tumour	2.43 (2.09 - 2.83)	7.83 (6.03 - 10.16)	1.33 (1.08 - 1.64)
Any hematologic tumour	4.03 (3.73 - 4.35)	7.88 (6.70 - 9.28)	3.40 (3.10 - 3.72)
Leukemia	2.20 (1.53 - 3.18)	4.33 (2.06 - 9.11)	1.87 (1.22 - 2.88)
Lymphoma	2.03 (1.79 - 2.31)	5.38 (4.29 - 6.76)	1.37 (1.16 - 1.62)
Multiple myeloma	1.36 (1.04 - 1.78)	2.80 (1.76 - 4.45)	1.04 (0.74 - 1.47)
Other hematologic tumour	13.51 (11.72 - 15.58)	31.61 (21.22 - 47.08)	11.81 (10.12 - 13.79)

© 2021 Giannakeas V et al. *JAMA Network Open*.

eFigure 1. Flow diagram of inclusion cohort and exclusion criteria.

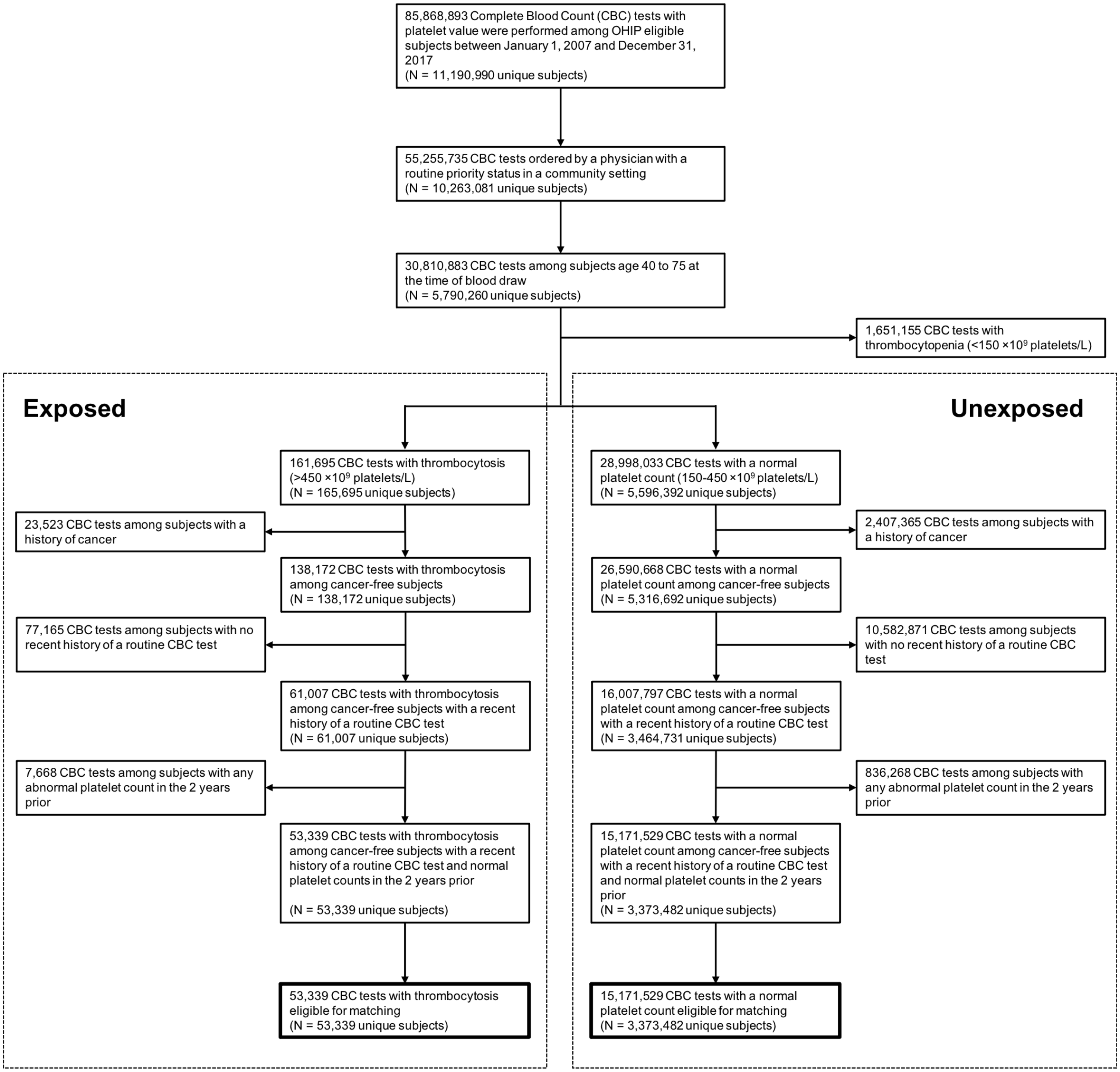

© 2021 Giannakeas V et al. *JAMA Network Open*.

eFigure 2. Cancer incidence rates by week of follow-up among thrombocytosis patients and matched controls (primary analysis).

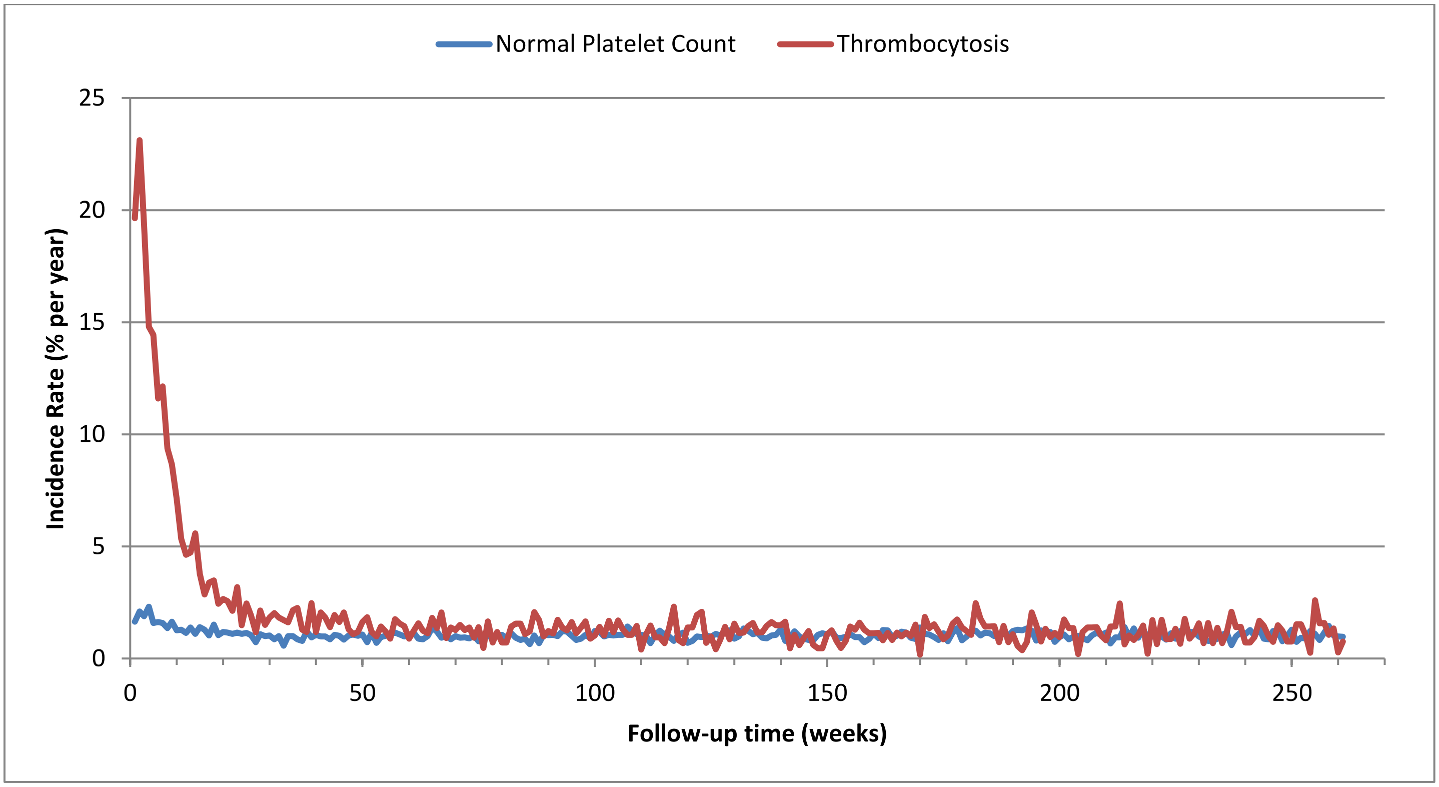

© 2021 Giannakeas V et al. *JAMA Network Open*.

eFigure 3. Relative risks for the development of incident cancer for those with thrombocytosis compared to those with a normal platelet count, by time elapsed since blood test and by cancer site (cervix, endometrium, bladder, and pancreas).

	Thrombocytosis		Normal Platelet Count			
	Cancer	At Risk	Cancer	At Risk	P-value	Estimate [95% CI]
Cervix						
0 to 6 months	12	36,384	17	181,920	0.0008	3.53 [1.69, 7.39]
>6 to 12 months	*S*	34,744	*S*	180,113	0.8923	0.86 [0.10, 7.18]
>1 to 2 years	*S*	34,213	*S*	178,653	0.3371	1.74 [0.56, 5.40]
>2 to 3 years	*S*	28,592	*S*	150,843	0.9448	1.06 [0.23, 4.82]
>4 to 5 years	*S*	18,714	*S*	100,111	0.7044	0.67 [0.08, 5.35]
Overall	20	36,384	63	181,920	0.0718	1.59 [0.96, 2.62]
Endometrium						
0 to 6 months	48	36,384	88	181,920	<.0001	2.73 [1.92, 3.88]
>6 to 12 months	13	34,744	67	180,113	0.9846	1.01 [0.56, 1.82]
>1 to 2 years	22	34,213	110	178,653	0.8525	1.04 [0.66, 1.65]
>2 to 3 years	18	28,592	100	150,843	0.8400	0.95 [0.58, 1.57]
>3 to 4 years	13	23,397	73	124,168	0.8511	0.95 [0.52, 1.70]
>4 to 5 years	12	18,714	64	100,111	0.9923	1.00 [0.54, 1.86]
Overall	126	36,384	502	181,920	0.0224	1.25 [1.03, 1.53]
Bladder						
0 to 6 months	36	51,624	85	258,120	0.0002	2.12 [1.43, 3.13]
>6 to 12 months	21	48,888	44	255,064	0.0006	2.49 [1.48, 4.19]
>1 to 2 years	18	47,995	100	252,663	0.8334	0.95 [0.57, 1.57]
>2 to 3 years	16	40,002	96	212,924	0.6574	0.89 [0.52, 1.51]
>3 to 4 years	14	32,568	95	174,832	0.4130	0.79 [0.45, 1.39]
>4 to 5 years	12	25,919	73	140,327	0.7082	0.89 [0.48, 1.64]
Overall	117	51,624	493	258,120	0.0958	1.19 [0.97, 1.45]
Pancreas						
0 to 6 months	48	51,624	64	258,120	<.0001	3.75 [2.58, 5.45]
>6 to 12 months	*S*	48,888	*S*	255,064	0.4202	0.65 [0.23, 1.84]
>1 to 2 years	22	47,995	56	252,663	0.0039	2.07 [1.26, 3.39]
>2 to 3 years	9	40,002	54	212,924	0.7394	0.89 [0.44, 1.80]
>3 to 4 years	*S*	32,568	*S*	174,832	0.5803	1.19 [0.64, 2.23]
>4 to 5 years	13	25,919	38	140,327	0.0550	1.85 [0.99, 3.48]
Overall	108	51,624	298	258,120	<.0001	1.81 [1.45, 2.26]

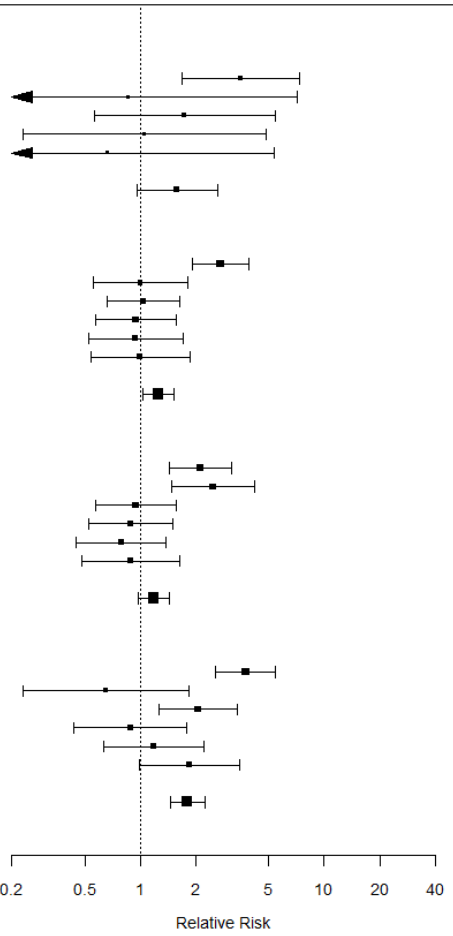

© 2021 Giannakeas V et al. *JAMA Network Open*.

eFigure 4. Relative risks for the development of incident cancer for those with thrombocytosis compared to those with a normal platelet count, by time elapsed since blood test and by cancer site (brain, head and neck, other solid tumour, and any hematologic tumour).

	Thrombocytosis		Normal Platelet Count			
	Cancer	At Risk	Cancer	At Risk	P-value	Estimate [95% CI]
Brain						
0 to 6 months	*S*	51,624	*S*	258,120	0.7964	1.14 [0.43, 3.00]
>6 to 12 months	*S*	48,888	*S*	255,064	0.5687	0.65 [0.15, 2.84]
>1 to 2 years	6	47,995	18	252,663	0.2329	1.75 [0.70, 4.42]
>2 to 3 years	*S*	40,002	*S*	212,924	0.0956	0.18 [0.03, 1.35]
>3 to 4 years	*S*	32,568	*S*	174,832	0.6485	1.34 [0.38, 4.76]
>4 to 5 years	*S*	25,919	*S*	140,327	0.6391	1.35 [0.38, 4.80]
Overall	20	51,624	109	258,120	0.7231	0.92 [0.57, 1.48]
Head and neck						
0 to 6 months	17	51,624	56	258,120	0.1318	1.52 [0.88, 2.61]
>6 to 12 months	11	48,888	29	255,064	0.0539	1.98 [0.99, 3.96]
>1 to 2 years	15	47,995	78	252,663	0.9652	1.01 [0.58, 1.76]
>2 to 3 years	13	40,002	55	212,924	0.4565	1.26 [0.69, 2.30]
>3 to 4 years	8	32,568	48	174,832	0.7707	0.89 [0.42, 1.89]
>4 to 5 years	8	25,919	31	140,327	0.3989	1.40 [0.64, 3.04]
Overall	72	51,624	297	258,120	0.1428	1.21 [0.94, 1.57]
Other solid tumour						
0 to 6 months	144	51,624	92	258,120	<.0001	7.83 [6.03, 10.16]
>6 to 12 months	24	48,888	67	255,064	0.0086	1.87 [1.17, 2.98]
>1 to 2 years	31	47,995	99	252,663	0.0151	1.65 [1.10, 2.47]
>2 to 3 years	20	40,002	114	212,924	0.7776	0.93 [0.58, 1.50]
>3 to 4 years	15	32,568	73	174,832	0.7293	1.10 [0.63, 1.92]
>4 to 5 years	19	25,919	75	140,327	0.2185	1.37 [0.83, 2.27]
Overall	253	51,624	520	258,120	<.0001	2.43 [2.09, 2.83]
Any hematologic tumour						
0 to 6 months	372	51,624	236	258,120	<.0001	7.88 [6.70, 9.28]
>6 to 12 months	136	48,888	153	255,064	<.0001	4.64 [3.68, 5.84]
>1 to 2 years	241	47,995	307	252,663	<.0001	4.13 [3.49, 4.89]
>2 to 3 years	168	40,002	264	212,924	<.0001	3.39 [2.79, 4.11]
>3 to 4 years	117	32,568	251	174,832	<.0001	2.50 [2.01, 3.12]
>4 to 5 years	105	25,919	203	140,327	<.0001	2.80 [2.21, 3.54]
Overall	1,139	51,624	1,414	258,120	<.0001	4.03 [3.73, 4.35]

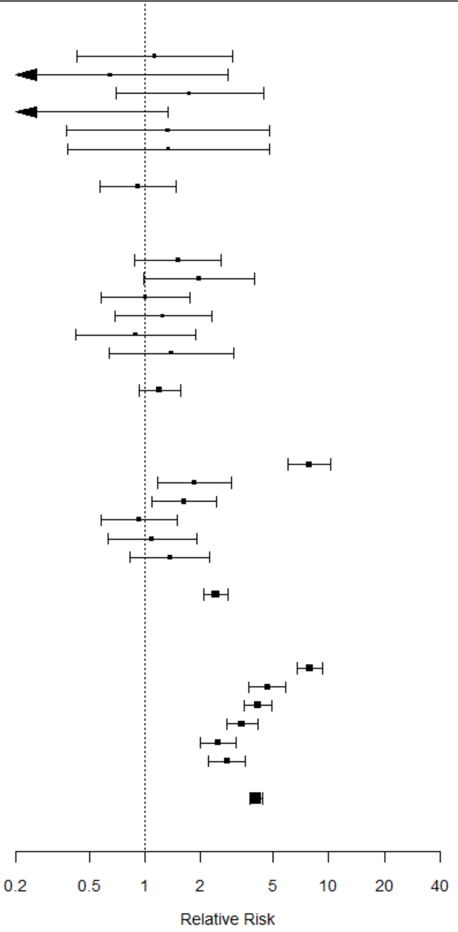

© 2021 Giannakeas V et al. *JAMA Network Open*.

eFigure 5. Relative risks for the development of incident cancer for those with thrombocytosis compared to those with a normal platelet count, by time elapsed since blood test and by cancer site (leukemia, lymphoma, multiple myeloma, and other hematologic tumour).

	Thrombocytosis		Normal Platelet Count			
	Cancer	At Risk	Cancer	At Risk	P-value	Estimate [95% CI]
Leukemia						
0 to 6 months	13	51,624	15	258,120	0.0001	4.33 [2.06, 9.11]
>6 to 12 months	*S*	48,888	*S*	255,064	0.0814	2.98 [0.87, 10.18]
>1 to 2 years	11	47,995	15	252,663	0.0007	3.86 [1.77, 8.40]
>2 to 3 years	6	40,002	22	212,924	0.4183	1.45 [0.59, 3.58]
>3 to 4 years	6	32,568	22	174,832	0.4078	1.46 [0.59, 3.61]
>4 to 5 years	*S*	25,919	*S*	140,327	0.4445	0.45 [0.06, 3.47]
Overall	41	51,624	93	258,120	<.0001	2.20 [1.53, 3.18]
Lymphoma						
0 to 6 months	154	51,624	143	258,120	<.0001	5.38 [4.29, 6.76]
>6 to 12 months	31	48,888	89	255,064	0.0042	1.82 [1.21, 2.73]
>1 to 2 years	52	47,995	173	252,663	0.0037	1.58 [1.16, 2.16]
>2 to 3 years	40	40,002	148	212,924	0.0412	1.44 [1.01, 2.04]
>3 to 4 years	27	32,568	143	174,832	0.9487	1.01 [0.67, 1.53]
>4 to 5 years	26	25,919	116	140,327	0.3723	1.21 [0.79, 1.86]
Overall	330	51,624	812	258,120	<.0001	2.03 [1.79, 2.31]
Multiple myeloma						
0 to 6 months	28	51,624	50	258,120	<.0001	2.80 [1.76, 4.45]
>6 to 12 months	8	48,888	38	255,064	0.8094	1.10 [0.51, 2.35]
>1 to 2 years	14	47,995	62	252,663	0.5590	1.19 [0.67, 2.12]
>2 to 3 years	6	40,002	33	212,924	0.9412	0.97 [0.41, 2.31]
>3 to 4 years	6	32,568	35	174,832	0.8508	0.92 [0.39, 2.19]
>4 to 5 years	6	25,919	32	140,327	0.9731	1.02 [0.42, 2.43]
Overall	68	51,624	250	258,120	0.0245	1.36 [1.04, 1.78]
Other hematologic tumour						
0 to 6 months	177	51,624	28	258,120	<.0001	31.61 [21.22, 47.08]
>6 to 12 months	93	48,888	19	255,064	<.0001	25.54 [15.59, 41.82]
>1 to 2 years	164	47,995	57	252,663	<.0001	15.15 [11.21, 20.47]
>2 to 3 years	116	40,002	61	212,924	<.0001	10.12 [7.43, 13.80]
>3 to 4 years	78	32,568	51	174,832	<.0001	8.21 [5.77, 11.68]
>4 to 5 years	72	25,919	43	140,327	<.0001	9.07 [6.21, 13.22]
Overall	700	51,624	259	258,120	<.0001	13.51 [11.72, 15.58]

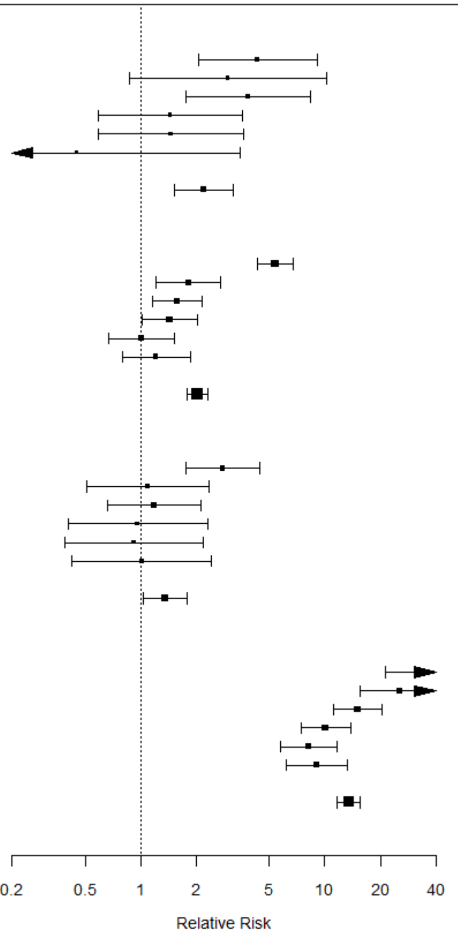

© 2021 Giannakeas V et al. *JAMA Network Open*.

References

1. Austin PC, van Walraven C, Wodchis WP, Newman A, Anderson GM. Using the Johns Hopkins Aggregated Diagnosis Groups (ADGs) to predict mortality in a general adult population cohort in Ontario, Canada. *Med Care.* 2011;49:932–939.
2. Gershon AS, Guan J, Wang C, To T. Trends in asthma prevalence and incidence in Ontario, Canada, 1996-2005: a population study. *Am J Epidemiol.* 2010;172:728–736.
3. Schultz SE, Rothwell DM, Chen Z, Tu K. Identifying cases of congestive heart failure from administrative data: a validation study using primary care patient records. *Chronic Dis Inj Can.* 2013;33:160–166.
4. Gershon AS, Wang C, Guan J, Vasilevska-Ristovska J, Cicutto L, To T. Identifying individuals with physcian diagnosed COPD in health administrative databases. *COPD.* 2009;6:388–394.
5. Jaakkimainen RL, Bronskill SE, Tierney MC, et al. Identification of Physician-Diagnosed Alzheimer's Disease and Related Dementias in Population-Based Administrative Data: A Validation Study Using Family Physicians' Electronic Medical Records. *J Alzheimers Dis.* 2016;54:337–349.
6. Antoniou T, Zagorski B, Loutfy MR, Strike C, Glazier RH. Validation of case-finding algorithms derived from administrative data for identifying adults living with human immunodeficiency virus infection. *PLoS One.* 2011;6:e21748.
7. Tu K, Campbell NR, Chen ZL, Cauch-Dudek KJ, McAlister FA. Accuracy of administrative databases in identifying patients with hypertension. *Open Med.* 2007;1:e18–e26.
8. Benchimol EI, Guttmann A, Mack DR, et al. Validation of international algorithms to identify adults with inflammatory bowel disease in health administrative data from Ontario, Canada. *J Clin Epidemiol.* 2014;67:887–896.
9. Lipscombe LL, Hwee J, Webster L, Shah BR, Booth GL, Tu K. Identifying diabetes cases from administrative data: a population-based validation study. *BMC Health Serv Res*. 2018;18:316.
10. Widdifield J, Bombardier C, Bernatsky S, et al. An administrative data validation study of the accuracy of algorithms for identifying rheumatoid arthritis: the influence of the reference standard on algorithm performance. *BMC Musculoskelet Disord.* 2014;15:216.
11. Mondor L, Maxwell CJ, Hogan DB, et al. Multimorbidity and healthcare utilization among home care clients with dementia in Ontario, Canada: A retrospective analysis of a population-based cohort. *PLoS Med.* 2017;14:e1002249.
12. Austin PC. Optimal caliper widths for propensity-score matching when estimating differences in means and differences in proportions in observational studies. *Pharm Stat*. 2011;10:150–161.

© 2021 Giannakeas V et al. *JAMA Network Open*.
